# Loose knots: Strong versus weak commitments to save for education in Uganda^☆^

**DOI:** 10.1016/j.jdeveco.2024.103444

**Published:** 2025-05

**Authors:** Dean Karlan, Leigh L. Linden

**Affiliations:** a The University of Texas at Austin, BREAD, IZA, J-PAL, NBER, United States; b Kellogg School of Management at Northwestern University, Global Poverty Research Lab, NBER, CEPR, United States

**Keywords:** Commitment savings, Micro-savings, Educational resources, School participation

## Abstract

Commitment devices offer an opportunity to restrict future choices. However, strict commitments may deter participation. Using a school-based commitment savings program for children to save for educational expenses in Uganda, we compare an account fully committed to school expenses to an account with a weaker commitment (funds withdrawn in cash, rather than a voucher). Children save more in the weaker commitment treatment arm, and when combined with parental outreach spend more on educational supplies and score 0.10 standard deviations (standard error = 0.04) on test scores. The fully committed account yields no such educational improvements, and neither account finds impacts on secondary or downstream outcomes such as attendance, enrollment, or non-cognitive skills.

## Highlights

• Tethered funds restrain.
• Yet weaker bonds free the mind—.
• Learning finds its path.
• And we learn that AI converts abstract to haiku-highlights quite well.

“Make it easy” – Richard Thaler, co-author of *Nudge: Improving Decisions about Health, Wealth, and Happiness* (Clement, 2013)

## 1 Introduction

A commitment device offers empowerment through restraint. Through such devices, a commitment-maker exercises their agency up-front in order to limit their range of future choices. In self-aware moments, individuals may choose to adopt these restrictions to resist future temptation or fend off social or filial pressures that are at odds with the commitment maker's goals. Indeed, prior research finds demand for commitment savings accounts that restrict access to one's money in order to help with self-control issues (Ashraf et al., 2006; Brune et al., 2016; Dupas and Robinson, 2013; Giné et al., 2018), and other research finds demand for commitment devices in other domains.

This project began after qualitative research on household finance in Uganda identified saving for school fees and supply costs as a key barrier for families.^1^ We tackle three primary questions within the context of an educational savings intervention. First, a program evaluation question: can a commitment savings program that encourages students to save improve student performance through increased educational expenditures? We discuss below but note this program offers not only a commitment savings account but also weekly opportunities to deposit following class discussions. Second, will the commitment savings account work better with a strict rule on how the accumulated funds are spent or a flexible rule? And third, does the savings-oriented commitment device change actual educational expenditures or instead does it get unwound through off-setting behavior?

The specifics of what one means by commitment on a commitment savings account varies considerably. For example, some accounts offer “hard” commitments that lock funds into a specific purpose, and others offer “soft” commitments such as merely labeling the purpose of the fund. The form and strictness of the commitment may each have different effects on each saving process step, such as account opening, deposits and withdrawals (size and frequency), and, perhaps most importantly, ultimate expenditure and investment decisions (see Amador et al. (2006) for a theoretical analysis of the overall tradeoff between commitment and flexibility).

Previous literature finds success across the spectrum of hard and soft commitment. In Kenya, Dupas and Robinson (2013) shows that soft savings commitment substantially induces people to invest in preventative health as compared to control. Also in Kenya, Duflo, Kremer, and Robinson (2011) finds hard commitments to save increased investment in fertilizers (specifically the hard commitment was in the form of a coupon to buy fertilizer). Steinert et al. (2022) tests two levels of soft commitment, and finds that a lockbox accompanied by a zip purse performed better than merely a lockbox (and the effects came from increased concealed resources from spouses rather than reduced temptation spending). In the United States, prior work has tested the impact of soft commitments as compared to hard commitments: Burke, Luoto, and Perez-Arce (2017) finds a hard commitment that restricted withdrawals to be more effective in increasing savings balances than soft commitments (non-binding pledge and a planning exercise). Similarly, in the United States, Beshears et al. (2020) finds that stronger commitment accounts (testing across several levels) that had the harshest level of early withdrawal penalty attracted more deposits.^2^

Drawing generalized claims about “soft” versus “hard” commitments is particularly challenging given the complexity of potential product features and the broad spectrum of contexts (health, agriculture, simple savings, e.g.). The lessons, we believe, will need to be more granular, i.e., focused on specific dimensions or features that have implications to the categorization into soft and hard.

In that spirit, we focus on one key dimension: whether the funds deposited are locked in for a specific “good” expenditure, or if individuals have the freedom to spend withdrawals as they wish in a setting in which the “good” item is made easily available.^3^ For this study, we define “hard” as one which locks spending in for specific purposes and “soft” as one which is labelled as such but not locked into such expenditures; however, we note that the terms “hard” and “soft” could also be referring to deposit requirements, costs to not complying with a plan, or other such tools to commit (see Bryan et al. (2010) for a broad discussion of types and evidence on commitment devices).

In theory the tradeoffs are clear: a strong commitment device may be more effective in enforcing the behavior of the future self, but the current self may be less likely to participate in the contract at all. An individual may want to commit in some, but not all, future states of the world, since emergencies do happen. The challenge is designing a contract in which a third party has the right level of enforcement discretion. This tension is highlighted in related work by John (2020), which argues that penalties for failing to complete commitments may be too weak for a certain range of the naïve, because the unfortunate case occurs too often (punishment for failure to complete the contract). If an individual cannot trust third parties with discretion to forgive without punishment, a self-enforcing commitment contract may instead be better. In such a contract, the increased price of vice is derived from psychic costs, i.e., disappointment with oneself and one's lack of adherence to a plan. This is akin to a model put forward by Benabou and Tirole (2004) on how personal rules can shift later behavior, and is a form of “mental accounting” (Shefrin and Thaler, 1992).

Our third question examines whether commitment devices get unwound through offsetting behavior (Karlan et al., 2014). More money spent from a commitment account for a particular purpose may simply crowd-out spending for that same purpose with funds from other sources. By examining how actual expenditures change for the particular purpose, rather than merely observing whether savings increases, we are able to make stronger statements about welfare-relevant outcomes, similar to Ashraf, 2010 with respect to household durable goods purchases and Dupas and Robinson (2013) with respect to health investments.

We examine these questions in the context of a school-based commitment savings account in Uganda for students roughly 12–14 years old. Specifically, we test whether a stronger versus a weaker savings commitment device helps children and their families save more, spend more on educational expenses, and achieve higher test scores. Relative to the economics of education literature, we thus gain a better understanding of the education production process (Kremer and Holla, 2009), building on a growing body of evidence demonstrating the possibly significant effects of basic school supplies – notebooks, uniforms, workbooks, etc. – on student performance (Das et al., 2013; Hidalgo et al., 2013) and parental involvement (Avvisati et al., 2013). Second, the results build on existing evidence of the importance of savings constraints for educational expenses (Barrera-Osorio et al., 2011) as well as mechanisms for tying resources to educational expenses (De et al., 2015).^4^

To implement the program, in collaboration with local schools in the Busoga sub-region of the Eastern region of Uganda, we worked with Private Education Development Network (PEDN) and Innovations for Poverty Action (IPA).

We randomly assigned 136 primary schools to one of three groups: (1) a strong commitment savings account (the “voucher” arm, in which funds could be withdrawn no earlier than the end of the term, and had to be spent on educational items through a voucher that we provided), (2) a weak commitment savings account (the “cash” arm, in which funds could be withdrawn no earlier than the end of the term, but were available in cash, to be spent as individuals wished),^5^ or (3) control. For both treatments, students could deposit cash into an account. At the end of each trimester, they were able to use their cash or vouchers to purchase school supplies at a fair.^6^ To facilitate the program and ensure teacher participation, we developed a brief teacher training component and also coordinated the transfer of money from a savings box held at the school to a local bank for safekeeping. One year into the implementation, after feedback from schools, we added a parental involvement workshop, randomly assigned to half of each of the two treatment arms.

The first stage is critical and revealing: students deposit significantly more money into the soft commitment savings account than the hard commitment savings account. And, for those with the parental outreach sub-treatment, the additional money deposited into the account leads to higher investment in school supplies, which then in turn leads to higher test scores. We find a 0.10 standard deviation (se = 0.04, 95%CI = [0.02, 0.18]) improvement in overall scores; this includes effects on each of the covered subjects: grammar (0.13 standard deviations, se = 0.04, 95%CI = [0.05,0.21]), reading (0.11 standard deviations, se = 0.05, 95% CI = [0.01,0.21]), and math (0.01 standard deviation, se = 0.05, 95%CI = [−0.09, 0.11]). The implication for the school production function is simple: for a student to learn basic skills, having a pen, paper, and workbook matters. Furthermore, the treatment effect on educational outcomes is sizable, as large as many direct educational interventions, and consistent with other estimates of the effects of such supplies (Das et al., 2013). We find no statistically significant effect on student participation (either attendance or enrollment) or on a set of non-cognitive outcomes.

One critical gap we leave in our understanding of the underlying mechanics: whose money went into the accounts, the child's or the parent's? Although the accounts were described as the students' accounts, we cannot rule out that some of the funds were considered parental funds and managed as such by the family.^7^ About half of our participating students reports engaging in some work and saving some from the money earned from work. This question muddles the ability to assert that the children (versus the parents) had time inconsistent preferences or if, on the other hand, the account shifted power across individuals with different preferences within the household. This is true, of course, in most studies on savings of individuals who live within a household. For example, in a typical “commitment savings” account test (e.g., see Ashraf et al., 2006; Dupas and Robinson, 2013; Brune et al., 2016), accounts are offered to individuals, and outcomes tracked at some combination of individual and household. Yet given fungibility of money within the household, it is difficult if not impossible to assert the source of the deposited funds. Because of the power dynamics between parent and child, there is a particular poignancy to this gap in our setting, yet the gap exists for any study of a savings intervention which targets individuals which live within a household of multiple adults.

## 2 Background

### 2.1 Ugandan primary education system

Uganda abolished most primary school fees in 1997.^8^ In the same year, the gross primary enrollment rate^9^ ballooned from 87 percent in the early 1990s, to 123 percent in 1997. Between 1996 and 1997, 2.3 million children enrolled in primary school, increasing total enrollment to 5.7 million (Murphy et al., 2002).

Unfortunately, while most children now enroll in primary school, the majority fail to graduate. In 2008, for example, the gross enrollment rate^10^ in lower secondary was 33 percent– 11 percentage points below the average for Sub-Saharan Africa (UNESCO, 2012). The transition from primary to secondary is a challenge, as in many countries. However, the majority do not complete primary school. As of 2010, only 32 percent of students entering primary school completed the seventh grade (UNESCO, 2012).

While the poor quality of primary education is a likely factor (Piper, 2010),^11^ students still face financial barriers. While students no longer pay enrollment fees, they do face other expenses. Many schools require uniforms, and families are responsible for providing food and school supplies, such as paper, writing instruments, and workbooks. See Appendix Table 1 for a summary of educational fees and expenses expected of households. With the approval of the parent-teacher association and school management committee, schools can also charge fees for ancillary services such as supplementary lessons, practice exams and feeding programs. Official policy prohibits preventing a child from enrolling due to an inability to pay, but the majority of dropouts cite financial concerns. In our baseline survey described below, families paid an average of 5790 UGX (2.30 USD) to send a child to school for a year, 0.5 percent of Uganda's per capita income in 2010 (UN data, 2013).

Confusion and suspicion create additional complications. As we discovered through qualitative interviews and feedback from parents, politicians try to drum up support by claiming school fees are illegal. The terms “universal” and “free” education are sometimes used interchangeably. Many parents do not understand the official financing rules. Some believe that the government should provide for all school related expenses. Finally, rumors of corruption can make even knowledgeable parents reluctant to pay.

### 2.2 Description of the intervention

To facilitate families' and children's saving for school, we evaluated four variations of a school-based savings program. The intervention had two primary objectives. First, it sought to facilitate and encourage the practice of children saving for education, and through saving, improve overall academic performance and support students' continued enrollment. The program targeted students in grades five, six and seven, i.e. the last three years of primary school, in order to target students at high risk for dropping out of school.^12^

We developed and implemented the programs in partnership with the Private Education Development Network (PEDN). PEDN is an Ugandan non-profit organization focusing on youth financial and entrepreneurial education. PEDN comprises five full and part time employees, often supplemented by project specific staff hired as needed. For the savings programs, IPA worked with PEDN to hire a local implementation team of about 10 people.^13^

Each treatment variation included the same core component: a savings account administered through the school, and a program to support and encourage children to use the accounts. During an introductory meeting, the implementation team described the program to a joint meeting of the Parent Teacher Association, the School Management Committee, and other interested parents. If they all voted to participate, we provided each school with metal lock boxes. A designated teacher assisted by student-elected^14^ representatives from each class then managed the program. The implementation team conducted weekly visits to each school to encourage saving and to assist with accounting procedures. Interested students received a passbook in which their individual savings were recorded, and the designated teacher and the implementation team maintained an official register. Depending on a school's preference, students then deposited money into the lockboxes on a daily or weekly basis.

To provide security and transparency, two padlocks secured each box. Parents elected a representative to keep the key to one lock, while the bank held the other. At the end of each trimester,^15^ the two key holders opened the box. The bank representative provided a deposit slip and deposited the funds into the school's account.^16^ The accounts did not earn interest. Inflation varied but averaged around 10% per year in this time period, thus the accounts had a negative real interest rate. After the break between trimesters, the implementation team and bank representatives returned to the school for the payout of the funds. Two representatives signed a withdrawal slip to confirm the withdrawal. The designated teacher, student representatives and our team then distributed the money according to the savings register. At the same time, the implementation team organized a small market at each school where students could purchase school supplies or school services such as practice exams or tutoring sessions (most of the funds went to school supplies, although detailed data are unfortunately not available).^17^

Thus, in net, treatment effects from the program (irrespective of variations discussed in a moment) compared to control schools could be a result of several factors. The commitment device is motivated by theories about time inconsistent preferences, but the weekly meetings also serve as a mere reminder to save. Reminders have been shown to generate higher savings, albeit on a sample of adults (Karlan et al., 2016). The meetings also were, in theory, informative, specifically teaching lessons about the importance of savings. In an evaluation of an in-school program to promote savings in Ghana, based on a program by Aflatoun, school children saved more in school but no downstream effects materialized on attitudes, aggregate savings, or education outcomes (Berry et al., 2018). One study is of course not dispositive, particularly given this is a different curriculum and setting, and thus it is important to note that the program could be shifting behavior due to the commitment aspect, attention, or information/signaling mechanisms.

On top of the core treatment above, there were four treatment variations, a 2x2 design: “cash” or “voucher” for the withdrawals, and “Parent Outreach” or “No Parent Outreach”.

For the cash treatment arm, students received, in cash, their savings from one trimester at the beginning of the next trimester. They could then spend the funds at their discretion—at the markets provided on the disbursement day (thus “making it easy” to spend on school supplies) or elsewhere. The voucher treatment arm, on the other hand, employed a stronger commitment — students had to buy educational products or services at the market, on the disbursement day.^18^ In both variants, children could also re-deposit their savings for the next trimester.

The Parent Outreach component was implemented halfway through the program as an adaptation based on qualitative feedback from teachers and schools. Specifically, there was demand from parents for more information about the program as well as incorrect beliefs being reported back to us via schools. Due to the potential importance and the relatively low cost of parent outreach, we randomized this component, implementing it for half of the treatment schools. The implementation team hosted a meeting for sixth and seventh grade parents. The meetings began by identifying the various stakeholders in primary education, their roles and responsibilities. PEDN then discussed the various ways in which parents could support their children's education. In particular, PEDN explained that in addition to providing a student learning experience, the savings program provided an opportunity for the household. It could be a tool to help families finance their children's education. A snack and soda were provided to encourage attendance.

## 3 Design of the evaluation

### 3.1 Research design

**Fig. 1:**
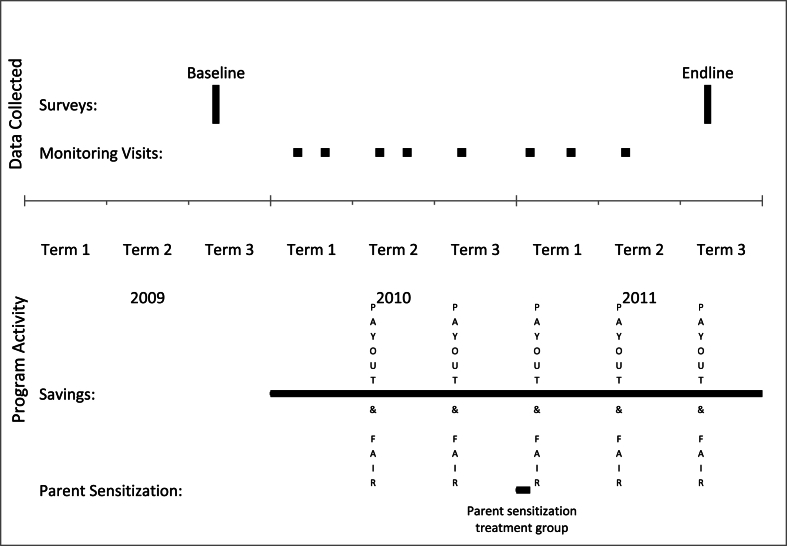
Research timeline.

Fig. 1 depicts the timeline for the randomized controlled trial and data collection. We selected 136 primary schools from the Jinja, Iganga, Mayuge, and Luuka districts of the Busoga Region because they predominantly comprised poor rural and peri-urban schools. We then administered a baseline survey and test during the final trimester of 2009. We then randomly assigned schools to receive either the cash treatment (39 schools), voucher treatment (39 schools), or no treatment (58 schools), stratifying by the total normalized score on the baseline exam and by geographic regions called sub-counties.^19^

Following the first randomization, school outreach began. It took two trimesters to recruit the majority of schools, but by the beginning of the third trimester of 2010, 95 percent of the treatment schools had agreed to participate (77 joined, 1 refused).^20^ The school that refused to participate did, however, permit data collection, and is thus included for all analysis in the treatment group.

In 2011, we conducted a second randomization for the parent sensitization program. To isolate the effect of the program while still treating all of the schools, we assigned schools either to the Parent Outreach group who received the intervention in the first trimester of 2011 or to the No Parent Outreach group who received the intervention too late to affect student behavior – immediately before the follow-up survey in second trimester. Half of the schools in each treatment were assigned to each group. We stratified assignment by the schools’ initial treatment group and sub-county, and checked for balance using the demeaned savings rates from 2010.

Finally, we conducted the follow-up survey and tests during the beginning of the third trimester of 2011.^21^

### 3.2 Data

Appendix Table 2 presents the specifics (year of survey, year of student, sample size) of the three datasets created.

We utilize two samples of students, as well as data at the classroom level. The “Attendance Survey” includes all students present in class at baseline and then tracks their attendance in subsequent rounds of data.

Second, we created a representative, longitudinal sample of students identified prior to treatment assignment (the “Student Survey”). These students were tracked regardless of whether or not they continued to be enrolled in the original schools.

The classroom-level data included all classes in grades five, six, and seven. Enumerators counted the number of children present, enrolled and possessing notebooks, math set, uniform, or shoes. 22^,^^23^ We conducted these monitoring visits prior to the randomization as part of the baseline and at least once a trimester after the randomization.

The Student Survey includes 4716 students who completed a baseline survey and aptitude test prior to the randomization. To identify the students for the second (longitudinal) student sample, we compiled a list of all students of the correct ages and grades in September of 2009 (P4 and P5, so that this constituted the students who would be in P5 and P6 for the start of the study).^24^ Teachers then classified each student using a five-point scale to rate frequency of attendance. In particular, this allowed us to identify students on the rosters who did not attend school. From the set of attending children, we randomly selected 35 students from each school, except for two schools in which we included all students because fewer than 35 students had enrolled.

The baseline survey completed by the students in the longitudinal sample was a 40 minute survey that included questions about their education history, experiences with saving, time preferences, and demographic information. Students also completed an hour-long, 35-question exam covering math, grammar, and reading comprehension. Students in each grade took separate exams based on the national curriculum for their grade.^25^

Students completed an follow-up survey about two years after the baseline survey. The 40 minute survey included questions about saving behavior, possession of resources like those in the class-level survey, such as uniforms, books, math sets, and shoes.

Tests were conducted at the same time as the baseline and follow-up surveys, for grammar, reading and math. Tests were about one hour long.^26^ We developed these tests ourselves, and aimed for them to mimic other exams they take in school. We piloted the tests to make sure we did not observe large modes at either the high or low extreme, and all analysis standardizes scores by subject relative to the contemporaneous control group.

We attempted to survey all students regardless of their enrollment status at the two-year follow-up, and successfully surveyed 3832 of the original respondents.

Finally, we verified the presence of each student in the longitudinal sample during each class-level monitoring visit. This provided an objective measure of students’ attendance rates as well as whether students were still enrolled in school in the appropriate grade.

Unfortunately, we lack two datasets which would have been fruitful, but were not feasible to collect: individual level savings data, and specific purchase decisions from the fairs in which school supplies were sold.

### 3.3 Orthogonality of treatment assignment and attrition

In Table 1, we verify the effectiveness of the randomization in creating observably similar treatment and control groups on average. Each row presents estimates for the indicated baseline characteristic. Columns 1–3 provide the sample size for each variable,^27^ the pooled treatment mean and standard deviation, and the control mean and standard deviation. Column 4 provides the regression estimates of the difference between the combined treatment group and control group, while Columns 5–8 provide regression estimates of the difference between each treatment group and the control group. All differences are estimated using equation (1) below, controlling for the sub-counties in which the schools were stratified.

**Table 1:** Summary Statistics and Orthogonality Verification of Random Assignment, Full Sample Frame from Baseline Mean (standard deviation) and OLS.

Dependent Variables	Number of Obs.	Mean (std dev)		OLS (one specification per cell)	OLS (one specification per row)				P-value for test of Cash Parent = Other Treatments
		Any Treatment	Control	Any Treatment	Cash with Parent Outreach	Voucher with Parent Outreach	Cash w/o Parent Outreach	Voucher w/o Parent Outreach	
	(1)	(2)	(3)	(4)	(5)	(6)	(7)	(8)	(9)
Classroom Survey: % of students in attendance	811	0.09	0.10	−0.01	−0.02	−0.01	−0.01	−0.01	0.55
		(0.16)	(0.18)	(0.01)	(0.01)	(0.01)	(0.01)	(0.01)	
				[0.79]	[1.00]	[1.00]	[1.00]	[1.00]	[1.00]
Classroom Survey: Supplies Index	813	0.03	0.01	0.02	0.22∗	0.04	−0.12	−0.05	0.02
		(1.06)	(1.01)	(0.10)	(0.11)	(0.15)	(0.19)	(0.14)	
				[1.00]	[0.77]	[1.00]	[1.00]	[1.00]	[0.32]
Normalized Test Score: Grammar	4710	0.08	−0.00	0.11	0.12	−0.02	0.18∗	0.14	0.87
		(0.99)	(1.00)	(0.07)	(0.11)	(0.09)	(0.10)	(0.09)	
				[0.79]	[1.00]	[1.00]	[0.77]	[1.00]	[1.00]
Normalized Test Score: Reading	4713	−0.00	−0.00	0.01	−0.02	−0.00	0.02	0.05	0.59
		(1.02)	(1.00)	(0.07)	(0.10)	(0.09)	(0.09)	(0.11)	
				[1.00]	[1.00]	[1.00]	[1.00]	[1.00]	[1.00]
Normalized Test Score: Math	4715	0.00	−0.00	0.02	−0.01	−0.07	0.07	0.08	0.72
		(0.98)	(1.00)	(0.06)	(0.09)	(0.08)	(0.10)	(0.10)	
				[1.00]	[1.00]	[1.00]	[1.00]	[1.00]	[1.00]
Normalized Test Score: Total	4716	0.03	−0.00	0.06	0.04	−0.03	0.11	0.11	0.77
		(1.00)	(1.00)	(0.07)	(0.11)	(0.09)	(0.10)	(0.11)	
				[1.00]	[1.00]	[1.00]	[1.00]	[1.00]	[1.00]
Student Survey: Attendance Code (lower = more attendance)	4716	1.43	1.42	0.00	−0.07	0.10	−0.01	0.00	0.33
		(0.96)	(0.90)	(0.07)	(0.12)	(0.12)	(0.11)	(0.10)	
				[1.00]	[1.00]	[1.00]	[1.00]	[1.00]	[1.00]
Student Survey: Days missed per school term	3886	1.63	1.64	−0.02	−0.04	−0.06	−0.07	0.10∗	0.57
		(0.91)	(0.95)	(0.04)	(0.07)	(0.07)	(0.07)	(0.06)	
				[1.00]	[1.00]	[1.00]	[1.00]	[1.00]	[1.00]
Student Survey: Prefer 500 Shillings today to 800 Shillings tomorrow	4702	0.65	0.64	0.01	0.02	−0.00	−0.04	0.07∗∗	0.83
		(0.48)	(0.48)	(0.02)	(0.04)	(0.03)	(0.03)	(0.03)	
				[1.00]	[1.00]	[1.00]	[1.00]	[0.77]	[1.00]
Student Survey: Prefer 500 Shillings today to 800 Shillings next week	4699	0.29	0.24	0.04∗∗	0.06	0.07∗∗	−0.02	0.07∗∗	0.68
		(0.45)	(0.43)	(0.02)	(0.04)	(0.03)	(0.03)	(0.03)	
				[0.79]	[1.00]	[0.77]	[1.00]	[0.77]	[1.00]
Student Survey: Child receives pocket money from family	4678	0.75	0.74	0.01	−0.02	0.03	0.07∗∗∗	−0.02	0.04
		(0.43)	(0.44)	(0.02)	(0.02)	(0.03)	(0.02)	(0.03)	
				[1.00]	[1.00]	[1.00]	[0.15]	[1.00]	[0.33]
Student Survey: Amount received in pocket money (shillings)	4698	204.20	214.45	−7.92	−17.84	−1.30	9.69	−21.83	0.30
		(266.36)	(297.52)	(13.91)	(16.31)	(19.61)	(18.07)	(19.39)	
				[1.00]	[1.00]	[1.00]	[1.00]	[1.00]	[1.00]
Student Survey: Primary use of money earned is school supplies	1983	0.23	0.27	−0.04	−0.03	−0.02	−0.07∗∗	−0.03	0.89
		(0.42)	(0.44)	(0.02)	(0.03)	(0.04)	(0.03)	(0.04)	
				[0.79]	[1.00]	[1.00]	[0.77]	[1.00]	[1.00]
Joint Significance Test f-stat: one regression per column with column header as dependent variable (p-value)				1.35	1.16	1.08	1.25	1.10	
				(0.21)	(0.32)	(0.38)	(0.27)	(0.37)	

Standard errors are in "()" brackets. Westfall-Young-adjusted p-values are in "[]" brackets. % of students in attendance: The enumerators count of students present during a classroom visit, divided by class enrollment provided by the teacher. Supplies Index: the normalized mean of 4 binary measures: whether a student has a uniform, notebook, mathset, and shoes. Attendance Code: A subjectively recorded code given with the enrollment data that indicates how frequently a student attends, from 1 (always attends) to 6 (never attends). OLS specifications: Columns 4 and Colums 5–8 include robust standard errors, clustered by school (the unit of randomization), and subcounty fixed effects (the stratification variable). Column 9 is the p-value of an F-test of sigificance on a regression of the cash parent treatment against all other treatments and the same specifications as in Columns 5–8. UGX = Ugandan Shillings, 1 USD = 2815 UGX. ∗p < 0.10 ∗∗p < 0.05 ∗∗∗p < 0.01.

Overall, the differences are minimal, i.e., the assignment to each treatment is orthogonal to a series of baseline variables. Of the 83 estimates presented, nine are statistically significant: one at the one-percent level, five at the five percent level, and three at the ten percent level. The overall joint test of significance presented in the bottom row is not significant for any treatment group. Most importantly, the magnitudes of the estimated differences are also all relatively small. Regardless, the main specification includes control for baseline value of the outcome variables as well as test scores. In the appendix we then repeat the main tables without these additional controls, and results are qualitatively similar, suggesting that any imbalance at baseline was due to measurement error.

Table 2 analyzes attrition. First, Row 1 presents the basic test for whether treatment led to differential attrition rates overall. Columns 2 and 3 show that we have similar survey completion rates in treatment and control (82 and 81 percent), and Row 1 Columns 5–8 report no differences in attrition rates across the four treatment groups. However, even though the overall attrition rate is not affected by assignment to treatment, differential attrition could result in differences in the analysis sample frame (i.e., those who complete the follow-up survey, or take the follow-up exams). To test for this, we replicate Table 1 analysis on various baseline measures (rows 2 onward). The table is organized similarly to Table 1 (except that the classroom variables are omitted, since there is no attrition at the classroom level). Overall, we find no evidence of compositional effects from differential attrition. Only six tests are statistically significant (out of 66), and the only differences from Table 1 are the estimates for days missed per school term and the time preference measures.

**Table 2:** Summary Statistics and Orthogonality Verification of Random Assignment, Post-Attrition Sample Frame Mean (standard deviation) and OLS.

Dependent Variables	Number of Obs.	Mean (std dev)		OLS (one specification per cell)	OLS (one specification per row)				P-value for test of Cash Parent = Other Treatments
		Any Treatment	Control	Any Treatment	Cash with Parent Outreach	Voucher with Parent Outreach	Cash w/o Parent Outreach	Voucher w/o Parent Outreach	
	(1)	(2)	(3)	(4)	(5)	(6)	(7)	(8)	(9)
Follow-up Survey Completed (of Baseline Students)	4716	0.82	0.81	0.00	0.01	0.00	−0.01	0.02	0.74
		(0.39)	(0.39)	(0.01)	(0.02)	(0.02)	(0.02)	(0.02)	
				[1.00]	[1.00]	[1.00]	[1.00]	[1.00]	[1.00]
Normalized Test Score: Grammar	3832	0.09	0.01	0.11	0.13	−0.02	0.19∗∗	0.14	0.81
		(0.99)	(1.00)	(0.07)	(0.11)	(0.09)	(0.09)	(0.10)	
				[1.00]	[1.00]	[1.00]	[1.00]	[1.00]	[1.00]
Normalized Test Score: Reading	3835	0.01	0.01	0.02	−0.00	0.01	0.02	0.06	0.66
		(1.02)	(1.00)	(0.07)	(0.11)	(0.09)	(0.09)	(0.12)	
				[1.00]	[1.00]	[1.00]	[1.00]	[1.00]	[1.00]
Normalized Test Score: Math	3837	−0.00	0.01	0.02	0.01	−0.07	0.05	0.07	0.85
		(0.98)	(0.98)	(0.06)	(0.09)	(0.09)	(0.10)	(0.10)	
				[1.00]	[1.00]	[1.00]	[1.00]	[1.00]	[1.00]
Normalized Test Score: Total	3837	0.04	0.01	0.06	0.06	−0.02	0.10	0.10	0.87
		(0.99)	(0.99)	(0.07)	(0.11)	(0.09)	(0.09)	(0.11)	
				[1.00]	[1.00]	[1.00]	[1.00]	[1.00]	[1.00]
Student Survey: Attendance Code (lower = more attendance)	3837	1.42	1.42	−0.02	−0.08	0.07	−0.04	−0.02	0.39
		(0.94)	(0.90)	(0.07)	(0.12)	(0.12)	(0.10)	(0.10)	
				[1.00]	[1.00]	[1.00]	[1.00]	[1.00]	[1.00]
Student Survey: Days missed per school term	3145	1.62	1.63	−0.01	−0.03	−0.04	−0.06	0.08	0.56
		(0.91)	(0.93)	(0.05)	(0.07)	(0.08)	(0.07)	(0.06)	
				[1.00]	[1.00]	[1.00]	[1.00]	[1.00]	[1.00]
Student Survey: Prefer 500 Shillings today to 800 Shillings tomorrow	3824	0.65	0.65	0.00	0.01	0.00	−0.06∗	0.06	0.92
		(0.48)	(0.48)	(0.02)	(0.04)	(0.04)	(0.03)	(0.04)	
				[1.00]	[1.00]	[1.00]	[1.00]	[1.00]	[1.00]
Student Survey: Prefer 500 Shillings today to 800 Shillings next week	3821	0.29	0.25	0.04∗	0.05	0.06∗	−0.01	0.06∗	0.67
		(0.45)	(0.43)	(0.02)	(0.04)	(0.03)	(0.03)	(0.03)	
				[1.00]	[1.00]	[1.00]	[1.00]	[1.00]	[1.00]
Student Survey: Child receives pocket money from family	3805	0.75	0.74	0.01	−0.01	0.02	0.06∗∗	−0.02	0.19
		(0.43)	(0.44)	(0.02)	(0.02)	(0.03)	(0.02)	(0.03)	
				[1.00]	[1.00]	[1.00]	[1.00]	[1.00]	[1.00]
Student Survey: Amount received in pocket money (shillings)	3821	199.30	217.59	−15.15	−18.53	−12.75	5.05	−32.81∗	0.66
		(248.80)	(303.02)	(15.12)	(18.68)	(21.49)	(18.94)	(17.66)	
				[1.00]	[1.00]	[1.00]	[1.00]	[1.00]	[1.00]
Student Survey: Primary use of money earned is school supplies	1647	0.22	0.26	−0.04	−0.03	−0.02	−0.08∗∗∗	−0.02	0.77
		(0.41)	(0.44)	(0.02)	(0.03)	(0.04)	(0.03)	(0.04)	
				[1.00]	[1.00]	[1.00]	[0.35]	[1.00]	[1.00]

Standard errors are in "(.)" brackets. Westfall-Young-adjusted p-values are in "[.]" brackets. % of students in attendance: The enumerators count of the number of students present during a classroom visit, divided by the enrollment in the class as provided by the teacher. Supplies Index: the normalized mean of 4 binary measures: whether a student has a uniform, notebook, mathset, and shoes. The coefficient is expressed as standard deviations from the control mean. Attendance Code: A subjectively recorded code given with the enrollment data that indicates how frequently a student attends, from 1 (always attends) to 6 (never attends). OLS specifications: Columns 4 and 5–8 include robust standard errors, clustered by school (the unit of randomization), and subcounty fixed effects (the stratification variable). Column 9 is the p-value of an F-test of sigificance on a regression of the cash parent treatment against all other treatments and the same specifications as in Columns 5–8. UGX = Ugandan Shillings, 1 USD = 2815 UGX. ∗p < 0.10 ∗∗p < 0.05 ∗∗∗p < 0.01.

## 4 Results

Since the random assignment should ensure the orthogonality of treatment assignment and other student characteristics, our primary specification estimates the treatment effects via ordinary least squares using the following specification:

(1) $$Y_{ijk}=\alpha +\tau ’treat_{j}+\delta ’X_{ijk}+\varepsilon_{ijk}\text{.}$$

The variable *Y*_*ijk*_ is the dependent variable of interest. We perform estimates at the student and class level. The index *i* then represents either the student or class in school *j* and sub-county *k*. The vector ***treat***_***j***_ is a vector of indicator variables for each treatment, and ***X***_***ijk***_ is a vector of control variables. For each estimate, we control for baseline test scores in math, reading, and grammar; sub-county fixed effects; and, the baseline value of the outcome if available. We cluster standard errors by the unit of randomization, the school.

The experimental design was registered in the American Economic Association registry in January 2014, slightly more than two years after the end of the experiment (the AEA registry did not begin until 2013). As such, we have no prespecified specification to prioritize. Given that, we choose to present the most disaggregated specification of each of the four treatment arms without pooling as our core specification, but also show the p-values for each of the sub-treatment variations (cash versus voucher; parental outreach vs no parental outreach).

First, we assess students’ savings behavior. In Table 3, we provide two measures of total program savings over the two years: the total per school and per student (using three measures of the latter). Columns 1–4 provide the average for each research group. Focusing on the 2011 results, and with a less restrictive measure of the student body (attendance at any point during the two-year study period), the two cash payout treatment groups produce average per student savings of 3604 UGX and 2913 UGX in the parent outreach and no parent outreach groups, respectively. Using average attendance, these results approximately double to 4411 and 3672, respectively. In comparison, the two voucher treatments, with and without parent outreach, show average savings of 1262 UGX and 1511 UGX with a less restrictive measure of attendance; and 1595 UGX and 1772 UGX using average attendance. The differences between cash and voucher are statistically significant at the 1% level for average deposits per school and at the 5% level for average deposits per student for both measures of attendance (Column 5). On the other hand, the differences between parent and no parent outreach are not statistically significantly different from zero (Column 6). The results for 2010 (Panel A) are similar, albeit with smaller magnitudes.

**Table 3:** Super Savers Program Savings by Treatment Group in '000 UGX Mean (standard deviation).

	Mean (standard deviation)				P-value from *t*-test	
	Cash with Parent Outreach	Voucher with Parent Outreach	Cash w/o Parent Outreach	Voucher w/o Parent Outreach	Cash vs Voucher	Outreach vs. No Outreach
	(1)	(3)	(2)	(4)	(5)	(6)
**Panel A: 2010**						
Average Cumulative Deposits Made per School (2010)	180.29	109.09	186.76	105.24	0.02	0.95
	(232.49)	(84.84)	(126.37)	(86.44)	[0.04]	[0.93]
Average Cumulative Deposits Made per Student in 2010 (any attendance)	0.95	0.58	0.99	0.48	0.00	0.96
	(0.84)	(0.52)	(0.73)	(0.39)	[0.01]	[0.93]
Average Cumulative Deposits Made per Student in 2010 (avg attendance)	1.28	0.78	1.43	0.69	0.00	0.83
	(1.08)	(0.67)	(1.11)	(0.60)	[0.01]	[0.90]
Average Cumulative Deposits Made per Student (baseline attendance)	1.37	0.90	1.99	0.73	0.00	0.49
	(1.20)	(0.78)	(2.06)	(0.64)	[0.01]	[0.65]
**Panel B: 2011**						
Average Cumulative Deposits Made per School (2011)	346.78	156.78	366.47	185.07	0.00	0.59
	(357.38)	(71.03)	(225.81)	(128.67)	[0.01]	[0.76]
Average Cumulative Deposits Made per Student in 2011 (any attendance)	3.60	1.26	2.91	1.51	0.03	0.73
	(5.47)	(0.61)	(2.22)	(1.34)	[0.04]	[0.81]
Average Cumulative Deposits Made per Student in 2011 (avg attendance)	4.41	1.60	3.67	1.77	0.03	0.71
	(6.98)	(0.68)	(2.90)	(1.55)	[0.04]	[0.81]
Average Cumulative Deposits Made per Student (baseline attendance)	2.79	1.33	3.47	1.41	0.00	0.48
	(2.39)	(0.84)	(2.22)	(1.48)	[0.01]	[0.65]
Number of Schools	19	19	20	20		

Standard errors are in "(.)" brackets. Westfall-Young-adjusted p-values are in "[.]" brackets. Results from bank administrative school-level data. Note that these data are collected at the school level, i.e., the Average Deposits per Student is the average across schools of the average deposits per student at each school. Number of students per school is calculated using the attendance data from 5 visits in the first year and 3 visits in the second year. The "any attendance" specification counts any student who attended during any of the visits; the "avg attendance" uses the average number of students present over the visits. OLS specifications: Columns 5–6 include subcounty fixed effects (the stratification variable). UGX = Ugandan Shillings, 1 USD = 2815 UGX. ∗p < 0.10 ∗∗p < 0.05 ∗∗∗p < 0.01.

We draw three conclusions from the savings data. First, the more restrictive savings vehicle, the voucher treatment, generated significantly less savings than the less restrictive cash treatment. Second, for those in either of the savings treatment groups, we find no additional effect of the parent outreach on savings (and the parental outreach treatment was only implemented within the treatment groups, not within the control group, thus we can estimate its treatment effect in an environment with the savings treatments). This supports the upcoming evidence that while the cash treatment arm led to higher savings, the parent outreach component shifted *how* the funds were spent.

Table 4-A, Table 4-BC examines other key process and intermediate outcomes. First, in Panel A, we examine further savings outcomes as reported by students in the follow-up survey. We find that 79 percent of treatment students and only 11 percent of control students were familiar with the Supersavers program. Similarly, 44 percent of treatment group students and only 3 percent of control group students reported saving with Supersavers. There was little difference in program awareness or self-reported participation on the extensive margin across treatment groups. This thus supports the argument that the difference in outcomes is not due to differential marketing or promotion of the program, or differential compliance to experimental protocols, but rather to the attractiveness of the cash versus voucher condition and the parent outreach. We also observe an increase in self-reported in-school savings, but a larger reduction in self-reported out-of-school savings (large enough to lead to a negative impact on total reported savings, whether winsorized or not). We do not understand the negative estimate for total impact and posit that this could be a measurement or reporting issue or could be a by-product of different patterns of depositing and withdrawing into home versus school savings vehicles. The question asks about flow (albeit vaguely). Savings at school were not available for withdrawal, hence money deposited remained on deposit until the end of term. Savings at non-school vehicles likely could be withdrawn frequently, rotating funds in and out for petty trade, for instance. The ideal would have been to have a record of the average daily balance, but we do not do that, and in reflection we are not sure we would have been able to elicit this without considerable measurement error (particularly given time constraints of the survey).

**Table 4-A:** Process Outcomes, Self-Reported in Follow-up Survey, Intent to Treat Estimates Mean (standard deviation) and OLS.

Dependent Variables	Number of Obs.	Mean (std dev)		OLS (one specification per cell) Any Treatment	OLS (each row = one regression)				P-value for test of Cash w/Parent = Other Treatments	P-value for test of Cash w/Parent = Voucher w/Parent
		Any Treatment	Control		Cash w/Parent Outreach	Voucher w/Parent Outreach	Cash w/o Parent Outreach	Voucher w/o Parent Outreach		
	(1)	(2)	(3)	(4)	(5)	(6)	(7)	(8)	(9)	(10)
**Panel A: Savings Process Outcomes (Self-reported in Student Follow-up Survey - 2011)**										
Heard of Super Savers Program	3823	0.79	0.11	0.67∗∗∗	0.69∗∗∗	0.67∗∗∗	0.68∗∗∗	0.64∗∗∗	0.24	0.77
		(0.41)	(0.32)	(0.02)	(0.02)	(0.03)	(0.02)	(0.03)	[1.00]	[1.00]
				[0.00]	[0.00]	[0.00]	[0.00]	[0.00]		
Ever Talked with Parents about Saving?	3821	0.51	0.36	0.15∗∗∗	0.15∗∗∗	0.13∗∗∗	0.17∗∗∗	0.16∗∗∗	0.77	0.71
		(0.50)	(0.48)	(0.02)	(0.03)	(0.03)	(0.03)	(0.03)	[1.00]	[1.00]
				[0.00]	[0.00]	[0.00]	[0.00]	[0.00]		
Saved with Super Savers	3824	0.44	0.03	0.40∗∗∗	0.42∗∗∗	0.39∗∗∗	0.44∗∗∗	0.35∗∗∗	0.41	0.49
		(0.50)	(0.18)	(0.02)	(0.02)	(0.04)	(0.03)	(0.04)	[1.00]	[1.00]
				[0.00]	[0.00]	[0.00]	[0.00]	[0.00]		
Ever Saves Money	3821	0.79	0.79	0.01	0.01	0.02	0.02	−0.02	0.87	0.38
		(0.40)	(0.41)	(0.02)	(0.02)	(0.03)	(0.03)	(0.02)	[1.00]	[1.00]
				[0.45]	[0.67]	[0.42]	[0.61]	[0.39]		
Primary Source of Savings was Work	3830	0.43	0.47	−0.03∗	−0.06∗∗	−0.04	0.01	−0.04∗∗	0.23	0.59
		(0.50)	(0.50)	(0.02)	(0.03)	(0.03)	(0.03)	(0.02)	[1.00]	[1.00]
				[0.12]	[0.09]	[0.34]	[0.67]	[0.08]		
Any Funds Saved Came from Work	3822	0.49	0.53	−0.03∗	−0.04	−0.04	0.00	−0.04∗∗	0.71	0.97
		(0.50)	(0.50)	(0.02)	(0.03)	(0.03)	(0.03)	(0.02)	[1.00]	[1.00]
				[0.12]	[0.22]	[0.24]	[0.76]	[0.09]		
Any Funds Saved Came from Pocket Money from Parents Relative or Non-relative	3830	0.39	0.37	0.01	0.02	0.01	0.01	−0.00	0.50	0.88
		(0.49)	(0.48)	(0.02)	(0.03)	(0.02)	(0.03)	(0.03)	[1.00]	[1.00]
				[0.45]	[0.49]	[0.62]	[0.70]	[0.75]		
Any Funds Saved Came from Parents for the Purpose of Saving	3822	0.05	0.02	0.03∗∗∗	0.03∗∗∗	0.05∗∗∗	0.03∗∗	0.03∗∗	0.88	0.42
		(0.22)	(0.13)	(0.01)	(0.01)	(0.01)	(0.01)	(0.01)	[1.00]	[1.00]
				[0.00]	[0.01]	[0.00]	[0.07]	[0.09]		
Number of Locations Actively Used for Savings	3830	0.86	0.79	0.08∗∗∗	0.09∗∗∗	0.10∗∗	0.07∗	0.07∗∗	0.71	0.48
		(0.61)	(0.52)	(0.02)	(0.03)	(0.04)	(0.04)	(0.03)	[1.00]	[1.00]
				[0.00]	[0.01]	[0.04]	[0.12]	[0.03]		
Savings Attitude Index	3830	0.05	−0.00	0.06	0.04	0.11	0.06	0.02	0.77	0.21
		(1.00)	(1.00)	(0.04)	(0.08)	(0.07)	(0.07)	(0.06)	[1.00]	[1.00]
				[0.21]	[0.62]	[0.20]	[0.41]	[0.67]		
Student Spent Savings on School Fees or Supplies or Lunch	3816	0.47	0.40	0.07∗∗∗	0.09∗∗∗	0.11∗∗∗	0.05∗	0.04	0.43	0.18
		(0.50)	(0.49)	(0.02)	(0.03)	(0.03)	(0.03)	(0.03)	[1.00]	[1.00]
				[0.00]	[0.00]	[0.00]	[0.10]	[0.27]		
Total Self-Reported Savings Last Term	3830	7029.00	7878.30	−634.65	127.79	−1493.61∗∗	8.60	−1129.61∗	0.22	0.20
		(13994.37)	(15095.04)	(490.68)	(826.65)	(733.24)	(714.66)	(616.46)	[1.00]	[1.00]
				[0.21]	[0.72]	[0.09]	[0.76]	[0.12]		
Total Self-Reported Savings Last Term (wins. 95%)	3830	5808.42	6606.29	−676.49∗∗	−459.51	−1263.00∗∗∗	−7.38	−941.18∗∗	0.49	0.56
		(8040.78)	(8338.76)	(277.72)	(448.15)	(427.14)	(453.77)	(367.13)	[1.00]	[1.00]
				[0.03]	[0.38]	[0.01]	[0.76]	[0.03]		
In-school Self-Reported Savings Last Term (wins. 95%)	3830	560.77	62.52	492.58∗∗∗	525.57∗∗∗	415.89∗∗∗	632.91∗∗∗	405.29∗∗∗	0.51	0.19
				[0.00]	[0.00]	[0.00]	[0.00]	[0.00]		
Out-of-school Self-Reported Savings Last Term (wins. 95%)	3830	4932.84	6465.81	−1391.98∗∗∗	−1245.07∗∗∗	−1727.81∗∗∗	−1027.29∗∗	−1547.86∗∗∗	0.66	0.57
		(7955.66)	(8289.77)	(283.64)	(471.75)	(440.35)	(445.95)	(380.41)	[1.00]	[1.00]
				[0.00]	[0.03]	[0.00]	[0.05]	[0.00]		

Standard errors are in "(.)" brackets. Westfall-Young-adjusted p-values are in "[.]" brackets. Savings Attitude Index includes 7 statements scored on a Likert scale, 1–5. Higher = more positive attitude toward saving. 1) Saving money is not necessary if you live at home with your family. 2) Saving is a good thing to do. 3) Saving is for adults only. 4) My parents or relatives would be proud of me for saving. 5) Managing to save makes me feel happy with myself. 6) It's better to spend money today than to save it for use in the future. 7) Every time I get money I put away some money for saving. In School and Out of School Self Reported Savings (Follow-up Survey): sum of amount of money respondents reported saving during the last school term in each location (at home in local bank, hidden at home, give to a family member, savings program at school -- which likely includes the savings held as part of the treatment, in a bank account of a family member, other). Due to outliers in the treatment group, especially in the Cash- No Parent group, these two variables are winsorized at the 95% level. The # of locations variable is censored at three because the survey did not record more than three; only 5 students (0.1% of sample) reported saving in three locations. OLS specifications: Columns 3 amd 4–8 include robust standard errors clustered by school (the unit of randomization); controls for students' baseline test scores in grammar, reading, and mat; if available, a control for the baseline value of the dependent variable; and subcounty fixed effects (the stratification variables). Column 9 (10) is the p-value of an F-test of sigificance on a regression of the cash w/parent treatment against all other treatments (the voucher w/parent treatment) and the same specifications as in Columns 5–8. UGX = Ugandan Shillings, 1 USD = 2815 UGX. ∗p < 0.10 ∗∗p < 0.05 ∗∗∗p < 0.01.

**Table 4-BC:** Process and Intermediate Outcomes, Intent to Treat Estimates Mean (standard deviation) and OLS.

Dependent Variables	Number of Obs.	Mean (std dev)		OLS (one specification per cell)	OLS (each row = one regression)				P-value for test of Cash Parent = Other Treatments	P-value for test of Cash w/Parent = Voucher w/Parent
		Any Treatment	Control	Any Treatment	Cash w/Parent Outreach	Voucher w/Parent Outreach	Cash w/o Parent Outreach	Voucher w/o Parent Outreach		
	(1)	(2)	(3)	(4)	(5)	(6)	(7)	(8)	(9)	(10)
**Panel B: Intermediate Outcomes (Classroom Visits)**										
School Supplies Index 2010	813	−0.16	−0.10	−0.08	0.11	−0.06	−0.15	−0.22	0.05	0.24
		(1.18)	(0.89)	(0.12)	(0.12)	(0.20)	(0.22)	(0.19)	[0.56]	[1.00]
				[0.40]	[0.40]	[0.67]	[0.57]	[0.30]		
School Supplies Index 2011	950	0.37	0.25	0.08	0.34∗∗	0.04	−0.09	0.04	0.01	0.13
		(0.91)	(0.89)	(0.11)	(0.13)	(0.18)	(0.19)	(0.15)	[0.18]	[1.00]
				[0.40]	[0.03]	[0.70]	[0.63]	[0.67]		
**Panel C: Other Process Outcomes (Self-reported in Student Follow-up Survey - 2011)**										
School Supplies Index	3830	0.00	0.00	−0.01	0.09∗	0.02	−0.10	−0.06	0.01	0.27
		(1.05)	(1.00)	(0.04)	(0.05)	(0.05)	(0.08)	(0.06)	[0.18]	[1.00]
				[0.45]	[0.14]	[0.67]	[0.27]	[0.37]		
School Fees (annual)	3525	28804.26	33580.77	−4816.38∗	−4104.35	−6298.36	−3909.25	−4940.97	0.83	0.67
		(64594.63)	(76629.27)	(2892.69)	(3328.13)	(3831.05)	(3820.30)	(3832.89)	[1.00]	[1.00]
				[0.12]	[0.29]	[0.16]	[0.38]	[0.27]		
Cost of Most Recent Test	2343	1506.71	1589.22	−61.12	−69.64	76.03	−300.42	26.49	0.95	1.00
		(2658.92)	(2843.68)	(188.32)	(273.16)	(256.64)	(242.67)	(298.73)	[1.00]	[1.00]
				[0.45]	[0.68]	[0.67]	[0.29]	[0.75]		
Parental Involvement Index	3830	0.01	0.00	0.00	0.03	−0.02	−0.00	−0.01	0.43	0.36
		(1.04)	(1.00)	(0.04)	(0.06)	(0.06)	(0.06)	(0.06)	[1.00]	[1.00]
				[0.51]	[0.62]	[0.68]	[0.76]	[0.75]		
Primarily Used Money Earned for School Fees or Supplies	3830	0.14	0.12	0.02∗	0.02	0.02	0.02	0.02	0.97	0.87
		(0.35)	(0.32)	(0.01)	(0.02)	(0.02)	(0.02)	(0.02)	[1.00]	[1.00]
				[0.10]	[0.27]	[0.27]	[0.34]	[0.40]		
Missed School Because Sent to Look for Fees or Lack of Scholastic Materials	3575	0.18	0.18	0.00	−0.01	0.00	0.02	−0.01	0.67	0.97
		(0.38)	(0.39)	(0.01)	(0.02)	(0.02)	(0.02)	(0.02)	[1.00]	[1.00]
				[0.51]	[0.67]	[0.76]	[0.39]	[0.66]		

Standard errors are in "(.)" brackets. Westfall-Young-adjusted p-values are in "[.]" brackets. School Supplies Index (Classroom Visits): Enumerators at several classroom visits each term counted the number of students with school supplies, which was then divided by the number of students in attendance. Component treatment effects reported in Appendix Table 4a. School Supplies Index (Follow-up Survey): a standardized index of the count of categories for which at least one item is owned of uniforms, notebooks, mathsets, and shoes. Components detailed in Appendix Table 4b. Parental Involvement Index includes 3 questions: 1) Student thinks parents are responsible for children's education 2) Has your parent come to your school in the past year? 3) Has your parent seen a report of yours from school in the past year? Component treatment effects reported in Appendix Table 4b. OLS specifications: Columns 4 amd 5–8 include robust standard errors clustered by school (the unit of randomization); controls for students' baseline test scores in grammar, reading, and math (not for Panel B since the dependent variable is school-level); if available, a control for the baseline value of the dependent variable; and subcounty fixed effects (the stratification variables). Baseline values are also included as controls for "School Supplies Index 2010″, "School Supplies Index 2011″, "School fees", and "Missed school because sent to look for fees or lack of scholastic materials" variables (others are not available at baseline). Column 9 (10) is the p-value of an F-test from a regression of the cash w/parent treatment against all other treatments and the same specifications as in Columns 5–8. UGX = Ugandan Shillings, 1 USD = 2815 UGX. ∗p < 0.10 ∗∗p < 0.05 ∗∗∗p < 0.01.

Next we examine process and attitude questions about savings, with particular attention on the parental outreach sub-treatment. Starting with savings attitudes, we do not observe changes in an index of seven questions.^28^ This may have implications for long-term change in saving behavior, if one posits that these attitudinal shifts are a necessary component for long-term behavior change, after the active involvement from the NGO and savings program. Alternatively, the measures may be flawed, or the attitudinal changes may be unnecessary; the learned pattern of savings may be possible to change without changing underlying savings attitudes (similar to one interpretation of the five-year financial education results in Horn et al. (2023)).

Continuing to try to unpack the source of savings changes, we examine a series of self-reported outcomes regarding source of funds and location of savings. We find a statistically significant but fairly small in magnitude increase in “Any Funds Saved Came from Parents for the Purpose of Saving” (from 2% to 5%, with similar shift across all four treatment arms) and similarly for number of locations of savings (0.79–0.86). 10.13039/100014337Furthermore, we find no difference in savings behavior for those in the parental outreach sub-treatment, thus reinforcing the mechanism interpretation that the parental outreach worked by shifting *how* saved funds got spent but did not shift by much the source or quantity of the savings.

In Panel B and C, we then examine intermediate outcomes, i.e., the possession of school supplies (measured both during classroom visits as well as in the follow-up survey^29^), parental involvement, and payment of school fees. Analyses of these questions aim to further help understand the mechanism through which the program worked. We present the results for each, but only find an impact on the possession of school supplies and whether students spent savings on school supplies, suggesting that the other mechanisms are not responsible for the observed impacts, or are poorly measured.

As an indicator of general spending on school related expenses, we collect data on school supplies observable to the survey both in the classroom and follow-up survey. Panel B presents the results on school supplies that could be observed during classroom visits. The classroom visit school supplies index is normalized with respect to the control group and takes the average of four proportions: proportion of students in the classroom possessing uniforms, notebooks, math sets, and shoes.^30^ In 2010, none of the treatment groups yields statistically significant increases relative to the control group, particularly after adjusting for multiple hypotheses.

For 2011, with an additional year of experience implementing the program and after the parent outreach had been fully launched, the Cash with Parent Outreach treatment arm performs considerably better than control, as well as the other three treatments (both when compared individually (0.34 standard deviation improvement), as well as when the other treatments are pooled with control, although after correcting for multiple hypotheses the p-values are 0.03 and 0.15, respectively). This result is then reinforced by the follow-up survey, reported in Panel C: The school supplies index from the self-reported survey also shows in Column 5 a 0.09 standard deviation improvement (se = 0.05, p-value = 0.09 unadjusted and 0.17 adjusted for multiple-hypotheses, with similar p-values of 0.01 and 0.15 for the tests comparing the Cash Parent arm to the other three arms).^31^ We do not however observe any statistically significant shifts in school fees expenditures (albeit with large standard errors), self-reported absence because of failure to pay school fees, or amount paid for most recent tests.^32^

Panel C reports on data from the follow-up survey on parental involvement and school outcomes. Although the school supplies and test score impacts are strongest on the Cash with Parent Outreach treatment cell, we do not observe a direct impact on an index of three questions^33^ regarding parental involvement in the child's education (or the individual components, as reported in Appendix Table 4b).

Next we turn to test score results in Table 5.^34^ We put forward two basic mechanisms here: first, the savings account enables the purchasing of school supplies that are necessary for learning; second, the parental outreach leads the households and children to use the savings accounts to actually spend the saved money on school supplies. This is consistent with the results in Table 4-A, Table 4-BC on the impact on school supplies. And likewise, this mechanism predicts that the Cash with Parent Outreach treatment group should generate the largest (or only) positive impacts. Column 5 indicates that Cash with Parent Outreach improves overall test scores by 0.10 standard deviations (se = 0.04, p-value = 0.02 and 0.10 when unadjusted and adjusted for multiple hypotheses, respectively). Looking at the components of the test, we find improvements in grammar (0.13 standard deviations with confidence interval = [0.01,2.25] at the 95% level after multiple hypothesis correction and reading (0.11 standard deviation, with confidence interval = [−0.2,0.24] at the 95% level)., but no effect on math. None of the other three treatment groups generates statistically significant improvements compared to the control group, either overall or for any subject. However, we do observe a 0.10 reduction in math test scores for the Voucher with Parent Outreach treatment group (p-value = 0.04 and 0.11 when unadjusted and adjusted for multiple hypotheses, respectively). While the magnitude of this negative effect is concerning, we make four points that lessen our concern: (1) the difference in aggregate test scores is not statistically significant (−0.02 standard deviations, se = 0.04), (2) the result is not statistically significant after multiple hypothesis correction, (3) the other two components are fairly precise null effects (0.02 and −0.00 standard deviations, se = 0.04 and 0.05), and (4) there is little reason to posit a differentially negative effect for math versus other subject areas.

**Table 5:** Effect of Super Savers on Normalized Test Scores, Follow-up 2011 Mean (standard deviation) and OLS.

	Number of Obs.	Mean (std dev)		OLS (one specification per cell)	OLS (each row = one regression)				P-value for test of Cash Parent = Other Treatments	P-value for test of Cash w/Parent = Voucher w/Parent
		Any Treatment	Control	Any Treatment	Cash w/Parent Outreach	Voucher w/Parent Outreach	Cash w/o Parent Outreach	Voucher w/o Parent Outreach		
	(1)	(2)	(3)	(4)	(5)	(6)	(7)	(8)	(9)	(10)
Grammar	3761	0.05	0.00	0.03	0.13∗∗∗	0.02	0.04	−0.06	0.00	0.06
		(1.05)	(1.00)	(0.03)	(0.04)	(0.04)	(0.05)	(0.06)	[0.01]	[0.05]
				[1.00]	[0.04]	[1.00]	[1.00]	[1.00]		
Reading	3758	0.02	0.00	0.01	0.11∗∗	−0.00	−0.04	−0.04	0.00	0.03
		(1.01)	(1.00)	(0.04)	(0.05)	(0.05)	(0.05)	(0.07)	[0.01]	[0.05]
				[1.00]	[0.10]	[1.00]	[1.00]	[1.00]		
Math	3761	−0.04	0.00	−0.05	0.01	−0.10∗∗	−0.01	−0.08	0.11	0.04
		(1.00)	(1.00)	(0.04)	(0.05)	(0.04)	(0.05)	(0.07)	[0.03]	[0.05]
				[1.00]	[1.00]	[0.11]	[1.00]	[1.00]		
Total	3758	0.01	−0.00	0.00	0.10∗∗	−0.02	−0.00	−0.06	0.00	0.02
		(1.02)	(1.00)	(0.03)	(0.04)	(0.04)	(0.04)	(0.07)	[0.01]	[0.05]
				[1.00]	[0.10]	[1.00]	[1.00]	[1.00]		

Standard errors are in "(.)" brackets. Westfall-Young-adjusted p-values are in "[.]" brackets. OLS specifications: Columns 4 and 5–8 include robust standard errors clustered by school (the unit of randomization); controls for students' baseline test scores in grammar, reading, and math; and subcounty fixed effects (the stratification variables). Column 9 (10) is the p-value of an F-test of sigificance on a regression of the cash parent treatment against all other treatments (the voucher w/parent treatment) and the same specifications as in Columns 5–8. ∗p < 0.10 ∗∗p < 0.05 ∗∗∗p < 0.01.

Interestingly, the positive test score results from the Cash with Parent Outreach treatment arm are consistent with Das et al. (2013) which finds similar effects resulting from a $3 per student increase in student supplies. Both sets of results contrast with the traditional view that resources have limited effects on learning (Kremer and Holla, 2009). Appendix Table 6 repeats Table 5 without the controls for baseline individual test scores, and while the coefficients are similar the standard errors (due to omitted control variables for baseline test scores) are higher, and only the grammar test result remains statistically significant.

We also examine whether the improved test scores arises through increased attendance or enrollment, but find no evidence for either. Table 6 Panel A reports results on observed attendance as well as an index of three self-reported questions on attendance, and Panel B reports results on enrollment. None of the treatments generates statistically significant improvements relative to the control group.^35^

**Table 6:** Effect of Super Savers on School Participation Mean (standard deviation) and OLS.

	Number of Obs. (1)	Mean (std dev)		OLS (one specification per cell)	OLS (each row = one regression)				P-value for test of Cash Parent = Other Treatments
		Any Treatment	Control	Any Treatment	Cash w/Parent Outreach	Voucher w/Parent Outreach	Cash w/o Parent Outreach	Voucher w/o Parent Outreach	
		(2)	(3)	(4)	(5)	(6)	(7)	(8)	(9)
**Panel A: Attendance Rate**									
2010	4707	0.34	0.35	−0.02	−0.06	−0.02	−0.03	0.02	0.22
		(0.42)	(0.42)	(0.03)	(0.04)	(0.04)	(0.04)	(0.04)	[0.83]
				[1.00]	[1.00]	[1.00]	[1.00]	[1.00]	
2011	4707	0.18	0.17	0.00	−0.02	0.00	0.02	0.01	0.38
		(0.36)	(0.35)	(0.02)	(0.03)	(0.03)	(0.03)	(0.02)	[0.83]
				[1.00]	[1.00]	[1.00]	[1.00]	[1.00]	
Overall (2010 & 2011 conbined)	4707	0.28	0.28	−0.01	−0.04	−0.01	−0.01	0.01	0.24
		(0.36)	(0.36)	(0.02)	(0.03)	(0.03)	(0.03)	(0.03)	[0.83]
				[1.00]	[1.00]	[1.00]	[1.00]	[1.00]	
Attendance Index	2926	−0.02	−0.00	−0.01	0.00	0.02	−0.05	−0.04	0.51
		(0.98)	(1.00)	(0.05)	(0.07)	(0.07)	(0.07)	(0.06)	
				[1.00]	[1.00]	[1.00]	[1.00]	[1.00]	[0.83]
**Panel B: Enrollment Rate**									
2010	4707	0.43	0.45	−0.03	−0.08	−0.03	−0.02	0.03	0.14
		(0.50)	(0.50)	(0.03)	(0.05)	(0.05)	(0.05)	(0.05)	
				[1.00]	[1.00]	[1.00]	[1.00]	[1.00]	[0.83]
2011	4707	0.22	0.22	−0.00	−0.03	−0.00	0.02	−0.01	0.34
		(0.41)	(0.41)	(0.02)	(0.04)	(0.03)	(0.04)	(0.03)	
				[1.00]	[1.00]	[1.00]	[1.00]	[1.00]	[0.83]

Standard errors are in "(.)" brackets. Westfall-Young-adjusted p-values are in "[.]" brackets. Attendance Rate: Based on a roll call of students on the official school enrollment list, counting only those students present in the class when roll call was done. Attendance Index: includes 3 self-reported questions on student attendance: 1) Of the five school days of last week, how many were you absent? 2) Think of a normal week from last term, of the five school days how many were you usually absent from school? 3) Think of a normal month from last term, how many days were you usually absent? Components detailed in Appendix Table 4d. Enrollment Rate: Based on teacher responses as to whether a student on the official school enrollment list, was still enrolled at that school. OLS specifications: Columns 4 and 5–8 include robust standard errors clustered by school (the unit of randomization); controls for students' baseline test scores in grammar, reading, and math; if available, a control for the baseline value of the dependent variable; and subcounty fixed effects (the stratification variables). Here, baseline values are available only for "Attendance Index" variable. Column 9 is the p-value of an F-test of sigificance on a regression of the cash parent treatment against all other treatments and the same specifications as in Columns 5–8 ∗p < 0.10 ∗∗p < 0.05 ∗∗∗p < 0.01.

Last we examine several attitudinal indices, and child labor, in Table 7. Starting with the five attitudinal indexes, we note caution in interpretation: in theory, these may be either intermediate outcomes influenced directly by the treatment(s), or consequences of the shift in resources and test scores. In practice, we have no statistically significant results after adjusting for multiple hypotheses.^36^

**Table 7:** Effect of Super Savers on Student Attitudes, Follow-up 2011 Mean (standard deviation) and OLS.

	Number of Obs.	Mean (std dev)		OLS (one specification per cell)	OLS (each row = one regression)				P-value for test of Cash Parent = Other Treatments
		Any Treatment	Control	Any Treatment	Cash w/Parent Outreach	Voucher w/Parent Outreach	Cash w/o Parent Outreach	Voucher w/o Parent Outreach	
	(1)	(2)	(3)	(4)	(5)	(6)	(7)	(8)	(9)
Self Esteem Index	3830	−0.02	−0.00	−0.03	−0.05∗∗	−0.03	−0.03	−0.01	0.25
		(0.44)	(0.44)	(0.02)	(0.02)	(0.02)	(0.03)	(0.03)	[1.00]
				[1.00]	[1.00]	[1.00]	[1.00]	[1.00]	
Time Preference Index	3820	2.05	2.07	−0.02	−0.02	−0.02	−0.00	−0.03	0.99
		(0.83)	(0.82)	(-0.02)	(0.04)	(0.04)	(0.04)	(0.04)	[1.00]
				[1.00]	[1.00]	[1.00]	[1.00]	[1.00]	
Locus of Control (binary)	3818	0.58	0.60	−0.02	−0.01	−0.02	−0.01	−0.03	0.74
		(0.49)	(0.49)	(-0.02)	(0.02)	(0.02)	(0.02)	(0.02)	[1.00]
				[1.00]	[1.00]	[1.00]	[1.00]	[1.00]	
Financial Independence Index	3830	−0.04	0.00	−0.03	−0.05	−0.13∗∗	0.06	−0.00	0.65
		(0.97)	(1.00)	(-0.03)	(0.06)	(0.06)	(0.06)	(0.05)	[1.00]
				[1.00]	[1.00]	[1.00]	[1.00]	[1.00]	
Aspiration Index	3830	−0.01	0.00	−0.03	−0.05	−0.03	0.02	−0.04	0.54
		(1.04)	(1.00)	(-0.03)	(0.06)	(0.06)	(0.04)	(0.06)	[1.00]
				[1.00]	[1.00]	[1.00]	[1.00]	[1.00]	
Total Annual Hours Worked (wins. 99%)	3830	295.33	294.96	6.88	1.01	−31.78	36.02	21.96	0.75
		(461.85)	(447.26)	(6.88)	(23.20)	(27.91)	(29.26)	(26.04)	[1.00]
				[1.00]	[1.00]	[1.00]	[1.00]	[1.00]	
Total Annual Income from Work (10k UGX) (wins. 99%)	3830	17.55	17.82	0.20	−1.50	−2.88	4.03∗	1.17	0.21
		(34.42)	(33.91)	(0.20)	(1.76)	(2.18)	(2.08)	(2.00)	[1.00]
				[1.00]	[1.00]	[1.00]	[1.00]	[1.00]	

Standard errors are in "(.)" brackets. Westfall-Young-adjusted p-values are in "[.]" brackets. Self Esteem Index: includes 10 statements each of which the student evaluated on a Likert scale, 1–5. All scales were converted after the fact so that higher on the scale meant higher self esteem. 1) I am satisfied with myself. 2) Sometimes I think I am no good at all. 3) I believe I have a number of good qualities. 4) I am able to do things as well as most children. 5) I do not have much to be proud of. 6) Sometimes I feel useless. 7) I believe I am a valuable person, at least as much as my classmates. 8) I wish I could have more respect for myself 9) I sometimes think that I am a failure. 10) When I think of myself, I usually think good thoughts. In addition to those 10 statements, there is one question: 11) Are you confident that you will be successful in the future? Components detailed in Appendix Table 4e. Time Preference Index: includes 2 hypothetical time preference choices. 1) Would you rather receive 500 shillings today or 800 shillings next week? 2) Would you rather receive 500 shillings today or 1000 shillings next week? From these, respondents were split into low, medium, and high future preference groups. Components detailed in Appendix Table 4f. Locus of Control: If a person is successful in life, is it because he or she was lucky or because he or she worked very hard? (1 = worked hard, 0 = lucky) Financial Independence Index: includes 3 questions: 1) How much money do you think you will get in the next 7 days? 2) How much money did you get in the past 7 days? 3) How much pocket money are you given to spend as you wish? Components detailed in Appendix Table 4f. Aspirations Index: includes 4 questions about academic and vocation aspirations: 1) If you graduate from primary school, will your life be better than if you hadn't graduated? 2) Do you think you will go to secondary school? 3) Do you think you will reach university? 4) What do you want to be when you grow up? (student responded with career that requires higher education) Components detailed in Appendix Table 4f. OLS specifications: Columns 4 amd 5–8 include robust standard errors clustered by school (the unit of randomization); controls for students' baseline test scores in grammar, reading, and math; if available, a control for the baseline value of the dependent variable; and subcounty fixed effects (the stratification variables). Here, Here, baseline values are available only for "Time Prefernce Index" variable. Column 9 is the p-value of an F-test of sigificance on a regression of the cash parent treatment against all other treatments and the same specifications as in Columns 5–8. UGX = Ugandan Shillings, 1 USD = 2815 UGX. ∗p < 0.10 ∗∗p < 0.05 ∗∗∗p < 0.01.

In terms of child labor, critics of financial education for youth posit that introducing children to savings and financial decision-making may have the unintended consequence of focusing their attention on income, and then discourage school attendance in order to work (Varcoe et al., 2005). Berry et al. (2018) tests this in Ghana with students of similar age as this study, and finds that a financial education curriculum along with a savings box (but no directive or facilitation of using the savings for education expenses) did lead to higher child labor, whereas if a social values component was added to the financial education curriculum, there was no impact on child labor. In our setting, we find no impact from the program on child labor, either hours worked or total wages. Overall, the estimates from Table 6, Table 7, combined with the other outcomes, indicate that the observed effects on learning occur through changes in available supplies rather than changes in attitude or participation.

Although we could examine whether individuals who saved more also experienced higher increases in test scores, we have no instrument for saving more beyond the experimental variation, and thus are unable to explore such a specification without ignoring endogeneity issues (i.e., that individuals who save more are also investing more in other ways to their education). Thus we do not explore such heterogeneity as part of our robustness tests.

To explore econometric robustness and robustness to risks of baseline imbalance, Appendix Tables 5 and 6 present the core results (Table 4-A, Table 4-BC–5), except without controls for baseline test scores. We find no changes in the core results.

## 5 Conclusion

Weaker rather than stronger commitments can yield stronger impacts on behavior change. In the context of an educational savings program, we find that families and children save more under a weaker commitment than a stricter commitment. The key difference was whether the funds had to be spent on educational expenses (strict) or were merely intended for the same (weak).

Broadly speaking, savings devices with withdrawal-side restrictions aim to help guide savers towards specific uses of their saved funds, such as education in the case here. This requires striking a balance, however: the account should have sufficient limitations to generate the desired actions (saving until a goal is reached, or spending saved money on a specific purpose), but not be so strict as to deter participation, deter savings, in the first place. Stricter limitations may backfire if individuals want some wiggle room in the case of emergency, some ability to adjust for the unknown.

Trust may also pose an issue for stricter commitment devices. For example, if the institution offering the account is also the vendor providing the goods to be purchased, the consumer must trust the institution to deliver quality products at reasonable prices, and not take advantage of a locked-in consumer. In our setup, the voucher (i.e., the stricter treatment) may have worked worse because individuals did not trust that proper and fairly-priced school supplies would be available. However, we ameliorated this by carefully working with the schools on the school fairs, and we also note that by the second year such issues of trust should have dissipated yet the treatment effect persists.

Parental engagement proved important for this savings and education program. When combined with a parent sensitization program, we find that families and children in the cash arm spend their savings on educational expenses (school supplies).^37^ This does not, however, alter school participation – we find no effects on enrollment or attendance – but does improve students' scores on grammar by 0.13 standard deviations and on reading by 0.11 standard deviations (and on aggregate test scores, include math, by 0.10 standard deviations). This suggests that financial constraints may play an important role in students' academic performance and that understanding the role of families’ financial decision process may be an important element in understanding the overall production process of education.

On a practical level, we consider several implementation issues important to explore. As a program designed to improve student learning, treatment effects of this magnitude are large compared to other evaluations of interventions designed to provide resources to schools or directly to children (Jameel Poverty Action Lab, 2014), but they are small relative to many other types of programs (most notably, for example, programs that provide additional resources while also changing pedagogical strategies). Taking the program's relatively low cost (2.24 USD per student per year) into account using the methodology proposed by Dhaliwal et al. (2014), however, the program delivers learning gains at a cost of 1.49 USD per tenth of a standard deviation or 6.71 standard deviations per 100 USD^38^ (note our estimates ignore the opportunity cost to the family of the alternative use of the funds saved). This is competitive relative to other programs. Relative to the 27 studies compared by J-PAL (2014), only four produce improvements in test scores more cost-effectively.

In terms of encouraging family savings, the program costs were high relative to the savings generated. However, if the program generated long term savings behavior change, then between the continued savings and the improvement in educational outcomes, it would surpass typical cost benefit calculations. Because we do not observe changes in attitudes, however, we cannot confidently predict that the long-term impacts will sustain themselves (although lack of attitude changes does not mean the results will not sustain themselves: attitudes are difficult to measure and may merely reflect noise, and furthermore the habit and pattern of saving could change and sustain without changing attitudes (e.g., see Horn et al., 2023)). On the cost side, it may be possible to reduce costs, particularly with implementation via mobile banking. This would obviate the need for physical transfer of cash to a bank and lower the risk of theft from keeping cash in a (albeit locked) box at the school. However, if the group nature of the intervention (i.e., the public and communal training) was an important element for take-up (through mimicking of or learning from peers) and adherence (through monitoring and potential for social recognition), then a mobile banking implementation may lose that visual classroom element. Although these peer mechanisms were not emphasized in the training and implementation of the program, the fact that the savings were done publicly may have had such an effect.

On a more theoretical level, these results open up many related questions. How does the optimality of looser versus stricter commitments depend on whether savings is long term or short-term? If one is saving for potentially short-run needs, such as a buffer stock, looser knots may be optimal; whereas long-term savings, such as for retirement, may require tighter commitments as the benefits from savings are too remote. Also with respect to timing, are external interventions of this sort effective in changing long term behavior, i.e., does the psychic cost of deviation persist, even without an outsider-led intervention?

Questions also persist regarding how such interventions influence intra-household dynamics. Did the intervention shift the preferences of the child, or the parents, or both, and what does this imply for intra-household cross-generational bargaining issues?

Lastly, design issues may be critical for such a program to work. For example, how critical was the timing element of the “soft” commitment device, i.e., the fact that the school supplies were immediately available for purchase at the time of withdrawal? If that was critical, it is a ringing endorsement for the “make it easy” mantra (much in the spirit of Thaler and Sunstein (2009)), and also implies that the soft commitment device may have worked for reasons elaborated on in Mullainathan and Shafir (2013), because it increased the attention of individuals to educational expenses at exactly the right moment, when they had cash in their hands.

## CRediT authorship contribution statement

**Dean Karlan:** Writing – review & editing, Writing – original draft, Visualization, Validation, Supervision, Resources, Project administration, Methodology, Investigation, Funding acquisition, Formal analysis, Data curation, Conceptualization. **Leigh L. Linden:** Writing – review & editing, Writing – original draft, Visualization, Validation, Supervision, Resources, Project administration, Methodology, Investigation, Funding acquisition, Formal analysis, Data curation, Conceptualization.

## Data Availability

Data will be made available on request.

## Footnotes

1 The initial role for the qualitative work was to collaborate with an insurance company and identify missing insurance products. Although the insurance work did not proceed, the dominance of low savings for school in the focus groups led the researchers to this project.

2 Naturally the soft vs. hard commitment tradeoff is relevant in other non-savings domains too, although few papers explicitly test variation on this dimension. Two exceptions: Spika et al. (2023) finds a hard commitment to exercise works considerably better than a soft commitment; Bettega et al. (2023) finds a hard commitment to limit gambling online leads to slightly less risk taking than a soft commitment.

3 Clearly in a perfect market, specifically one with zero transaction costs, this would make no difference: any items purchased with the locked-in commitment account could simply be sold in exchange for the most desirable item for the same value. In our market, supplies and services associated with primary education in Uganda, there are significant enough transaction costs to make such an exchange quite costly, and thus the original expenditure sticky.

4 It is interesting to note that, while we find that relaxing savings constraints improves educational outcomes, we find improvements in academic performance rather than participation. This contradicts the results of Barrera-Osorio et al. (2011) which finds that distributing funds at the time that families have to pay enrollment expenses improves enrollment rates. The difference may, in part, be due to the fact that unlike Uganda, Colombian schools still charged official fees for enrollment.

5 The weak commitment treatment arm is thus most similar to the SEED account in Ashraf et al. (2006), i.e., a commitment merely to not withdraw funds until a certain future point in time.

6 Note that control schools did not receive a fair. Thus, analysis of “any treatment” includes bundling the fairs as part of the treatment. However, we observe no positive treatment effect on school supplies for the voucher treatment arm compared to control, so we conclude that the fairs themselves did not have an observable direct treatment effect since their path to impact would, we believe, have been through the path of higher school supplies expenditures.

7 As we show below, both the children and other family members contribute to the accounts, raising the possibility that multiple household mechanisms are involved.

8 Initially, up to four children per family could attend school without paying tuition fees (Murphy et al., 2002).

9 The gross primary school enrollment ratio is the ratio of the number of enrolled primary school children, regardless of age, to the total number of primary school-aged children in the population.

10 The gross enrollment rate for lower secondary school is the ratio of the number of children enrolled in lower secondary school regardless of age relative to the total number of children in the population who are of age to attend secondary school.

11 The dramatic increases in enrollment have strained existing resources. In the average school in 2005, three children had to share the same textbook and 94 children crammed into a single classroom (Independent Evaluation Group, 2007).

12 Note that Uganda follows a 7 + 2+2 grade structure. Students attend primary school for seven years followed by two years each of lower and then upper secondary school. At baseline, the mean student age was 12 (sd dev = 1.52).

13 This includes only those individuals hired to implement the described programs. It does not include the research staff who conducted the surveys and monitoring visits described below.

14 The Ugandan educational system classifies children enrolled in primary school as “pupils” and those in secondary school as “students”. In this article, we refer to all enrolled children as students.

15 The academic year starts in February and follows a trimester system. Schools run for 12 weeks at a time. Students receive a three week break after the first and second terms, and schools are closed in December, January and February.

16 Working with the bank, FINCA Uganda, we designed an account for the intervention modeled on a traditional group savings account. We also provided the minimum 5000 UGX deposit and worked with the school's elected signatories to obtain the documentation required to open the accounts.

17 Students were allowed to rollover vouchers to future terms, and upon completion of the final year (P7), were allowed to withdraw any remaining balance in cash.

18 Early in the intervention there was concern that the teachers and community members mobilized to manage the supplies fair were marking up prices to take advantage of the situation. To avoid this, the supplies markets were taken over as part of the intervention. In collaboration with a wholesale distributer, prices were set to match typical market prices available to students, and the fairs were organized by the implementing NGOs directly. Managing the fairs as part of the intervention also ensured the essentials supplies were there. This does have implications for scale-up attempts, i.e., whether through explicit management or alternative approach, one likely needs to have a competitive market for supplies available for the students. From the success and volume of sales in the weak-commitment treatment arm, we infer that the mix of products was sufficiently diverse and pricing appropriate that lack of engagement in the strong-commitment treatment arm was not due to lack of trust or the wrong product mix, particularly given the repeat nature of the process.

19 In 2010, Uganda included four major jurisdictions called “regions.” Spread across the four regions, were 111 “districts.” Each district was divided into urban areas known as “municipalities” or rural areas called “counties.” Counties were further sub-divided into sub-counties. Depending on the population, a district could have as few as three or as many as thirty or more sub-counties.

20 When they were not canceled, meetings had to be held with school administrators, the school management committee, and the parent-teacher association for each school. Many were initially reluctant to hold additional meetings.

21 In 2012, we conducted a second, smaller experiment in which we randomly assigned a fraction of the original control group to receive the cash with sensitization program. We also collected the classroom-level data described below. However, the remaining control group proved too small. The point estimates are consistent with those presented here, but the standard errors are too large to provide meaningful information. These results are available upon request.

22 The enumerator only counted a student as having each item if the enumerator could see it.

23 Notebooks cost approximately 200 UGX (0.08 $USD) each. In Uganda, they are usually called “exercise books.” A math set costs approximately 1000 UGX (0.40 $USD) and includes such tools as a ruler, protractor and compass. Uniform and shoes each cost about 6000 UGX. (2.39 $USD) They are a traditional school requirement.

24 For a small number of classes, rosters were unavailable. We had to create a list of students based on the students present in class and information provided by the teacher.

25 For both the baseline and follow-up exams, all scores are normalized within grade and subject relative to the contemporaneous control distribution.

26 See Appendix for the exact questions used in the tests.

27 Sample sizes vary because subjects refused to respond to some questions.

28 Savings Attitude Index includes 7 statements each of which the student evaluated on a Likert scale, 1–5. All scales were converted after the fact so that higher on the scale meant more positive attitude toward saving. (1) Saving money is not necessary if you live at home with your family. (2) Saving is a good thing to do. (3) Saving is for adults only. (4) My parents or relatives would be proud of me for saving. (5) Managing to save makes me feel happy with myself. (6) It's better to spend money today than to save it for use in the future. (7) Every time I get money I put away some money for saving. Appendix Table 4b presents the results for each of the components in this index.

29 If control group households were buying school supplies earlier than treatment schools, because of the savings accounts and fairs, we would on average observe this because the classroom surveys were conducted during the term, not merely at the end of the term.

30 Appendix Table 4a presents the results for each of the components of this index.

31 Appendix Tables 4a, 4b and 4c provide the details for each component of the School Supplies Indices in Panels B and C. The differences seem to be driven primarily by exercise books, although the individual components analysis is less robust statistically.

32 This pattern of results is consistent with students' reports on the follow-up survey regarding the disposition of the saved funds. We observe that students in the cash treatment with the parent sensitization report spending 3.6 percent more of the saved funds on school related expenses than students in the cash treatment without the sensitization. This is not a large enough difference to explain our results, but we are skeptical of the accuracy of these self-reported answers. We observe no differences in the amount of the savings used to purchase food or clothing or given to other family members. The increase in school related expenditures primarily comes from “other” expenses. This difference, however, is likely too small to explain all of the observed increase in school supplies, suggesting that the parent sensitization functioned both to divert students' savings and other unsaved household resources towards school supplies (but only in the weak-commitment arm, i.e., the strong commitment rules dissuaded the parents – even if aware of the opportunity – from engaging in such reallocations). Ultimately however we recognize that these are small amounts and self-reported data and thus it is difficult to draw too much inference regarding the source of funds and any such reallocations.

33 The three questions in the Parental Involvement Index are (1) Student thinks parents are responsible for children's education (2) Has your parent come to your school in the past year? (3) Has your parent seen a report of yours from school in the past year? Appendix Table 4b presents the results for each of the components in this index.

34 Appendix Table 7 presents the correlations between baseline and follow-up test scores. Overall, test scores strongly correlate with each other, both across topics and across time.

35 Appendix Table 4d presents the results for the components of the index.

36 Appendix Tables 4e – 4f presents the results for the components of these indices.

37 Although we find that the voucher treatment led to about half the deposits as that of the cash arm, we do not find that school supplies increased by half. We posit two possible explanations. First, although the point estimate is close to zero, we cannot reject, statistically, a point estimate of half of that of the cash treatment effect. Second, the voucher treatment arm may have led to a reduction in school supplies through an anchoring effect (if the amount saved in vouchers was smaller for some than they would have spent otherwise).

38 Estimates are provided in 2011 USD.

## References

[bib1] Amador Manuel, Werning Iván, Angeletos George-Marios (2006). Commitment vs. Flexibility. Econometrica.

[bib2] Ashraf Nava (2010). “Female empowerment: further evidence from a commitment savings product in the Philippines.”. World Dev..

[bib3] Ashraf Nava, Karlan Dean, Yin Wesley (2006). Tying odysseus to the mast: evidence from a commitment savings product in the Philippines. Q. J. Econ..

[bib5] Avvisati Francesco, Gurgand Marc, Guyon Nina, Maurin Eric (2013). Getting parents involved: a field experiment in deprived schools. Rev. Econ. Stud..

[bib6] Barrera-Osorio Felipe, Bertrand Marianne, Linden Leigh, Perez-Calle Francisco (2011). Improving the design of conditional transfer programs: evidence from a randomized education experiment in Colombia. Am. Econ. J. Appl. Econ..

[bib7] Benabou Roland, Tirole Jean (2004). Willpower and personal rules. J. Polit. Econ..

[bib8] Berry James, Karlan Dean, Pradhan Menno (2018). The impact of financial education for youth in Ghana. World Dev..

[bib9] Bettega Paul, Crosetto Paolo, Dubois Dimitri, Romaniuc Rustam (2023). Hard vs. Soft commitments: experimental evidence from a sample of French gamblers. Working Paper. Grenoble Applied Economics Laboratory (GAEL).

[bib10] Brune Lasse, Giné Xavier, Goldberg Jessica, Yang Dean (2016). Facilitating savings for agriculture: field experimental evidence from Malawi. Econ. Dev. Cult. Change.

[bib11] Bryan Gharad, Karlan Dean, Nelson Scott (2010). Commitment devices. Annual Review of Economics.

[bib12] Clement Douglas (2013). Interview with richard thaler. Region.

[bib13] Das Jishnu, Dercon Stefan, Habyarimana James, Krishnan Pramila, Muralidharan Karthik, Sundararaman Venkatesh (2013). School inputs, household substitution, and teset scores. Am. Econ. J. Appl. Econ..

[bib14] De Arcangelis, Giuseppe, Joxhe Majlinda, McKenzie David, Tiongson Erwin, Yang Dean (2015). Directing remittances to education with soft and hard commitments: evidence from a lab-in-the-field experiment and new product take-up among Filipino migrants in rome. J. Econ. Behav. Organ..

[bib15] Dhaliwal Iqbal, Duflo Esther, Glennerster Rachel, Tulloch Caitlin, Glewwe Paul (2014). Education Policy in Developing Countries.

[bib16] Dupas Pascaline, Robinson Jonathan (2013). Why don't the poor save more? Evidence from health savings experiments. Am. Econ. Rev..

[bib17] Giné Xavier, Goldberg Jessica, Silverman Dan, Yang Dean (2018). Revising commitments: field evidence on the adjustment of prior choices. Econ. J..

[bib18] Hidalgo Diana, Onofa Mercedes, Oosterbeek Hessel, Ponce Juan (2013). Can provision of free school uniforms harm attendance? Evidence from Ecuador. J. Dev. Econ..

[bib19] Horn Samantha, Jamison Julian C., Karlan Dean, Zinman Jonathan (2023). Five-year impacts of group-based financial education and savings promotion for Ugandan youth. Rev. Econ. Stat..

[bib20] Independent Evaluation Group (IEG) (2007). http://www.worldbank.org/oed/education/uganda.html.

[bib21] Jameel Poverty Action Lab (2014). Student learning. http://www.povertyactionlab.org/policy-lessons/education/student-learning.

[bib22] John Anett (2020). When commitment fails: evidence from a field experiment. Manag. Sci..

[bib23] Karlan Dean, Ratan Aishwarya Lakshmi, Zinman Jonathan (2014). Savings by and for the poor: a research review and agenda. Rev. Income Wealth.

[bib24] Karlan Dean, McConnell Margaret, Mullainathan Sendhil, Zinman Jonathan (2016). Getting to the top of mind: how reminders increase saving. Manag. Sci..

[bib25] Kremer Michael, Holla Alaka, Cohen Jessica, Easterly William (2009). What Works in Development? Thinking Big and Thinking Small.

[bib26] Mullainathan Sendhil, Shafir Eldar (2013).

[bib27] Murphy Paul, Bertoncino Carla, Wang Lianqin (2002). Education Notes.

[bib28] Piper Benjamin (2010).

[bib29] Shefrin H., Thaler R. (1992). Choice over Time.

[bib30] Spika Devon, Wickström Östervall Linnea, Gerdtham Ulf, Wengström Erik (2023). Put a bet on it: can self-funded commitment contracts curb fitness procrastination?. https://ideas.repec.org//p/hhs/lunewp/2023_004.html.

[bib31] Steinert Janina Isabel, Satish Rucha Vasumati, Stips Felix, Vollmer Sebastian (2022). Commitment or concealment? Impacts and use of a portable saving device: evidence from a field experiment in urban India. J. Econ. Behav. Organ..

[bib32] Thaler Richard H., Sunstein Cass R. (2009).

[bib33] UN data (2013). United National Statistical Division.

[bib34] UNESCO (2012). Global Education Digest 2012.

[bib35] Varcoe Karen P., Martin Allen, Devitto Zana, Charles Go (2005). Using a financial education curriculum for teens. Journal of Financial Counseling and Planning.

