# Supplementary material for: Loose knots: Strong versus weak commitments to save for education in Uganda
Source: J Dev Econ. 2025 May;174:103444. doi: 10.1016/j.jdeveco.2024.103444 (PMC11894509; doi:10.1016/j.jdeveco.2024.103444)
Supplement: Multimedia component 2 [file mmc2.docx]

**Appendix: Baseline and Follow-up Aptitude Tests**

**Innovations for Poverty Action: Project 79**

**BASELINE Code**

**P4 Baseline Aptitude Test**

**TIME ALLOWED: 1 hr NAME:_________________________**

**SCHOOL: _________________________**

**DATE: ____________________________**

***Section 1: Mathematics***

1. What is the shaded fraction?

|  |  |  |  |  |
| --- | --- | --- | --- | --- |

__________________________________

1. 17 **3.** 8760  **4.** 375 **5.** 290

x 3 - 6420 + 250 x 5

______ __________ ________ ________

1. Deborah bought a dress at 5,300 /= and a pair of shoes at 2,000 /=. How much money did she spend?

____________/=

1. What is the time?


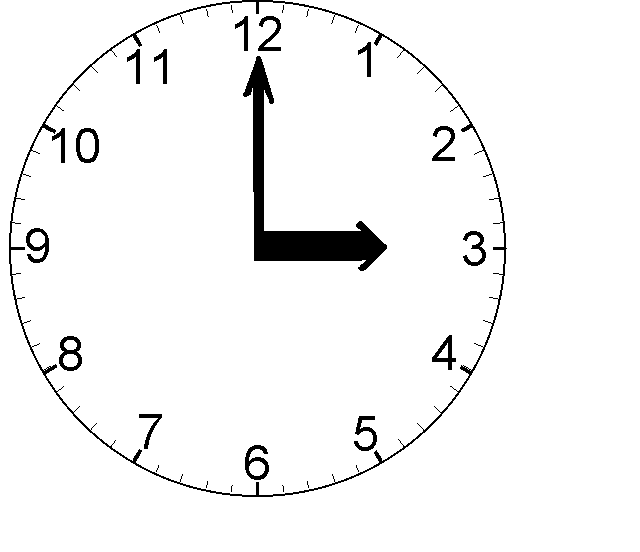
 ­­­__________________

1. 15 ÷ 3 = ____ ___ **9.** 8 – 10 = ____ __ _ **10.** 5 + -1 = ____ ___

**11.** ⅜ + ⅛ = ________ **12.** ⅞ – ⅝ = _______ __ **13.** 13 + 2.3 =__ ____

**14**. Calculate the distance around the triangle.


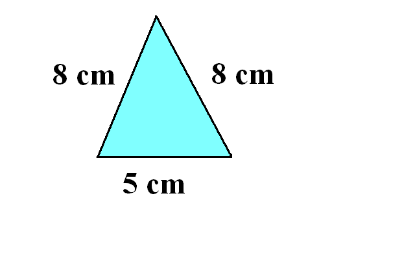
 Distance: ____________cm

***Section 2: English***

**IN QUESTIONS 15 AND 16, COMPLETE EACH SENTENCE WITH THE CORRECT FORM OF THE WORD IN BRACKETS.**

1. My sister always ­____________ to church on Sundays. (go)
2. I usually____________ up at 7:30 am. (wake)

**IN QUESTIONS 17 – 20, COMPLETE EACH WORD**

1. Bo__k **18.** Pen__il **19.** Tab__e **20.** S__hool

**IN QUESTIONS 21 AND 22 RE-WRITE EACH SENTENCE WITH THE OPPOSITE OF THE UNDERLINED WORD OR WORDS.**

1. Some objects sink in water.

________________________________________________________________

1. The floor is clean.

­­­­­­­­­­_______________________________________________________________

**IN QUESTIONS 23 AND 24, ARRANGE THE WORDS IN ALPHABETICAL ORDER.**

1. hot, egg, design, map

_________________________________________________________________

1. bridge, bat, beat

_________________________________________________________________

**USE THE FOLLOWING WORDS TO MAKE A SENTENCE:**

1. boy school a going to I saw

_ ____________________________

1. food good very restaurant The at this is

_____________________________

***Section 3: Reading Comprehension***

**STUDY THE INFORMATION BELOW AND ANSWER THE QUESTIONS**

**Budget for Juma’s Birthday Party on 29th November, 2009**

| **ITEM** | **QUANTITY AND UNIT** | **PRICE PER UNIT** | **TOTAL AMOUNT** |
| --- | --- | --- | --- |
| Chicken | 10 birds | 6,500 /= per bird | 65,000 /= |
| Meat | 10 kg | 3,000 /= per kg | 30,000 /= |
| Irish potatoes | 20 kg | 500 /= per kg | 10,000 /= |
| Cooking oil | 5 litres | 3,000 /= per litre | 15,000 /= |
| Charcoal | ½ sack | 20,000 /= per sack | 10,000 /= |
| Soda | 3 crates | 11,000 /= per crate | 33,000 /= |
| Cakes | 2 cakes | 300 /= per cake | 600 /= |

1. When is the party going to take place?

__________________________________________________________________

1. How many items are shown?

__________________________________________________________________

1. How much meat will be needed in quantity? __________________________________________________________________
2. What is the price per unit of one kg of Irish potatoes?

__________________________________________________________________

1. What is the total amount of money that will be spent on cooking oil? __________________________________________________________________
2. What is the most expensive item on the list in terms of price per unit? __________________________________________________________________
3. What quantity of charcoal will be bought? __________________________________________________________________

**READ THE STORY AND ANSWER THE QUESTIONS BELOW IN FULL SENTENCES.**

Sarah is a girl in P4. Yesterday was her sister’s wedding. Her whole family went to the party. Her sister had a beautiful white dress. She received many gifts. Our aunt gave her a nice mat. There was a lot of good food. Sarah ate some popcorn and some wedding cake. She did not eat any bread. There was a band and Sarah danced with her brother.

1. Which class is Sarah in?

…………………………………………………………………………………………

**37.** Who went to the party?

…………………………………………………………………………………………

**38.** What did Sarah eat?

…………………………………………………………………………………………

**39.** Who did Sarah dance with?

…..….………………………………………………………………………………….

**Innovations for Poverty Action: Project 79**

**BASELINE Code**

**P5 Baseline Aptitude Test**

**TIME ALLOWED: 1 hr NAME: ___________________________**

**SCHOOL: _________________________**

**DATE: ___________________________**

***Section 1: Mathematics***

1. A factory produces 290 bars of soap per day and works 5 days a week. How many bars of soap will the factory produce by the end of the week?

________________________ bars of soap

1. 15 ÷ 3 = ____ ___ **3.** 8 - 10 = ____ __ _ **4.** 5 + - 1 = __ __ ___

**5.** 17 **6.** 8760  **7.** 375 **8.** 290

x 3 - 6420 + 250 x 5

_____ _________ _______ ________

**9.** ⅛ + ½ = __________ **10.** ⅞ – ⅝ = _______ __

1. Sarah has two 1,000 /= notes. She wants change for the same amount. Which of the following is the correct change? Circle the correct answer.
   1. Three 200/= shilling coins, two 500/= coins and seven 100/= coins
   2. Five 200/= coins, four 500/= coins and six 100/= coins
   3. Two 500/= coins, two 200 coins and six 100/= coins
   4. Three 500/= coins, three 200/= coins and four 100/= coins

**12.** What is the time?


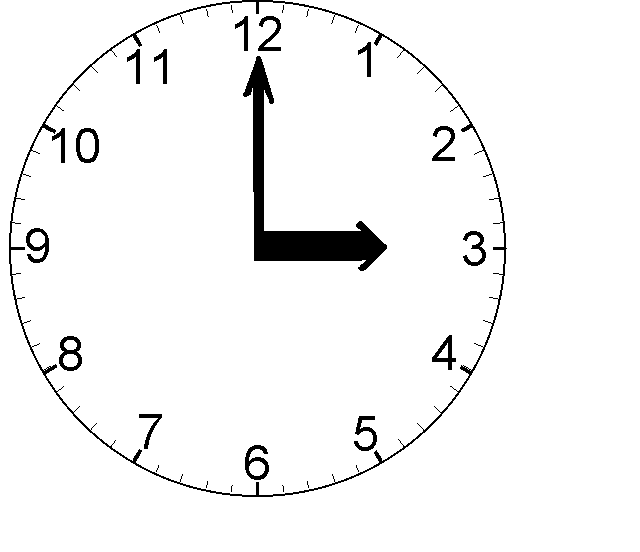
__________________

1. Calculate the perimeter of the figure.

8 cm

Perimeter: _____________

6 cm

6 cm

8 cm

***Section 2: English***

**IN QUESTIONS 15-17, COMPLETE EACH SENTENCE WITH THE CORRECT FORM OF THE WORD IN BRACKETS.**

1. There are pieces of _____________ glass on the floor. (break)
2. The teacher is not here, he has _____________ to town. (go)
3. My school bag is _____________ than yours. (heavy)

**IN QUESTIONS 17-18, RE-WRITE EACH SENTENCE WITH THE OPPOSITE OF THE UNDERLINED WORD.**

1. Some objects sink in water.

__________________________________________________________________

__________________________________________________________________

1. The floor is clean.

__________________________________________________________________

__________________________________________________________________

**IN QUESTIONS 19 AND 20, ARRANGE THE WORDS IN ALPHABETICAL ORDER.**

1. helicopter, egg, design, map

__________________________________________________________________

1. bridge, baby, booth, beat

__________________________________________________________________

**USE THE FOLLOWING WORDS TO MAKE A SENTENCE:**

1. boy school a going to I saw

_ ____________________________

1. food good very restaurant The at this is

_____________________________

***Section 3: English and Mathematics Combined***

**STUDY THE INFORMATION BELOW AND ANSWER THE QUESTIONS ABOUT IT IN FULL SENTENCES.**

**Budget for Juma’s Birthday Party on 29th November, 2009**

| **ITEM** | **QUANTITY AND UNIT** | **PRICE PER UNIT** | **TOTAL AMOUNT** |
| --- | --- | --- | --- |
| Chicken | 10 birds | 6,500 /= per bird | 65,000 /= |
| Meat | 10 kg | 3,000 /= per kg | 30,000 /= |
| Irish potatoes | 20 kg | 500 /= per kg | 10,000 /= |
| Cooking oil | 5 litres | 3,000 /= per litre | 15,000 /= |
| Charcoal | ½ sack | 20,000 /= per sack | 10,000 /= |
| Soda | 3 crates | 11,000 /= per crate | 33,000 /= |
| Cakes | 200 small cakes | 300 /= per small cake | 60,000 /= |
| Cakes | 5 big cakes | 30,000 /= per large cake | 150,000 /= |

1. When is the party going to take place?

_________________________________________________________________

1. How many items are shown?

_________________________________________________________________

1. How much meat will be needed in quantity?

_________________________________________________________________

1. What is the price per kg of Irish potatoes?

_________________________________________________________________

1. What is the total amount of money that will be spent on cooking oil? _________________________________________________________________
2. How many items will be purchased for a total amount of 10,000/= each?

_________________________________________________________________

1. What is the most expensive item on the list in terms of price per unit?

_________________________________________________________________

1. How much charcoal will be bought in quantity?

_________________________________________________________________

**USE THE INFORMATION IN THE PASSAGE BELOW TO ANSWER THE QUESTIONS THAT FOLLOW.**

Sarah has an older brother named Edward. Tomorrow, November 8^th^ is Edward’s birthday and Sarah has decided to bake him a cake. Today she will go to the market and buy the ingredients for her cake. Tomorrow, before Edward wakes up, she will mix the ingredients and bake the cake. The cake is going to be vanilla because it is Edward’s favorite flavor. Sarah prefers chocolate cakes but Edward doesn’t like chocolate.

Sarah has made a list of the ingredients that she needs in order to make her cake. She will go to the market to buy them.

The cost of each of the items was:

1. eggs: 3,000 /= for a bag of 6 eggs
2. milk: 500 /= for one bag
3. flour: 2,500 /= for one bag
4. sugar: 1,500 /= for one bag
5. baking powder: 3,000 /= for one tin
6. vanilla: 2,000 /= for one bottle
7. butter: 2,500 /= for one packet

**ANSWER THE FOLLOWING QUESTIONS BASED ON THE PASSAGE ABOVE. CIRCLE THE LETTER THAT CORRESPONDS TO THE CORRECT RESPONSE.**

31. Who is Edward?

a) Sarah’s younger brother

b) Sarah’s cousin

c) Sarah’s older brother

d) Sarah’s friend

32. What is Sarah going to do?

a) Buy a cake at the market

b) Buy a present for her brother

c) Play with her friend

d) Buy ingredients at the market and bake a cake

33. How much will Sarah spend on sugar and flour?

a) 3,500 /=

b) 3,000 /=

c) 300 /=

d) 4,000 /=

34. How much does a bag of six eggs cost?

a) 3,000 /=

b) 300 /=

c) 2,500 /=

d) 2,000 /=

35. If Sarah gave the shopkeeper a 5,000 /= note to pay for eggs, how much balance did the shopkeeper give back to Sarah?

a) 200 /=

b) 4,300 /=

c) 1,500 /=

d) 2,000 /=

36. What kind of cake is Sarah going to make?

a) Chocolate

b) Vanilla

c) Strawberry

d) Coffee

37. What kind of cake does Sarah prefer?

a) Chocolate

b) Vanilla

c) Strawberry

d) Coffee

38. If Sarah buys everything on her list except for sugar, how much will she spend?

a) 15,000 /=

b) 14,500 /=

c) 13,500 /=

d) 13,000 /=

**Innovations for Poverty Action: Project 79**

**A**

**Code**

**ID**

**FOLLOW UP EXAM**

**TIME ALLOWED: 1 hr NAME:__________________________**

**SCHOOL: __________________________**

**DATE: ____________________________**

***Section 1: Mathematics***

1. What is the shaded fraction?

|  |  |  |
| --- | --- | --- |

__________________________________

1. 15 **3.** 9650  **4.** 343 **5.** 290

x 2 - 4230 + 108 x 3

______ __________ ________ ________

1. Samuel bought a hoe for 6,000 /= and a packet of seeds for 3,500 /=. How much money did he spend in total?

____________/=

1. What is the time?


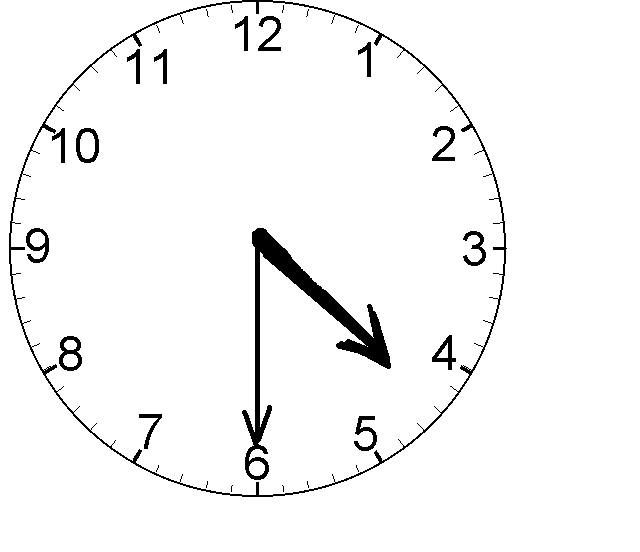
 ­­­__________________

1. 20 ÷ 4 = ____ ___ **9.** 6 – 8 = ____ __ _ **10.** 4 + -3 = ____ ___

**11.** ⅝ + ⅛ = ________ **12.** ⅔ - ⅓ = ______ __ **13.** 11 + 3.9 =__ ____

**14**. Calculate the distance around the rectangle.

7 cm

3 cm

Perimeter: ____________cm

***Section 2: English***

**IN QUESTIONS 15 AND 16, COMPLETE EACH SENTENCE WITH THE CORRECT FORM OF THE WORD IN BRACKETS.**

1. Yesterday we ­____________ some school supplies. (buy)
2. My older brother is much ____________ than I am. (tall)

**IN QUESTIONS 17 – 20, COMPLETE EACH WORD**

1. Hou_e **18.** L__ght **19.** Ga_den **20.** R_ad

**IN QUESTIONS 21 AND 22 RE-WRITE EACH SENTENCE WITH THE OPPOSITE OF THE UNDERLINED WORD OR WORDS.**

1. Some plants will live if they are put in bright sunlight.

________________________________________________________________

1. The store closes at 9 o’clock.

­­­­­­­­­­_______________________________________________________________

**IN QUESTIONS 23 AND 24, ARRANGE THE WORDS IN ALPHABETICAL ORDER.**

1. bucket, spent, nail, maize, house

_________________________________________________________________

1. return, rain, root, railway

_________________________________________________________________

**USE THE FOLLOWING WORDS TO MAKE A SENTENCE:**

1. we our for tomorrow study test will

_ ____________________________

1. very this raining morning started it early

_____________________________

***Section 3: Reading Comprehension***

**STUDY THE INFORMATION BELOW AND ANSWER THE QUESTIONS**

**Shopping List for Auma’s Stall at the Market**

| **ITEM** | **QUANTITY AND UNIT** | **PRICE PER UNIT** | **TOTAL AMOUNT** |
| --- | --- | --- | --- |
| Tomatoes | 10 kg | 2,500 /= per kg | 25,000 /= |
| Onions | 5 kg | 1,000 /= per kg | 5,000 /= |
| Irish potatoes | 20 kg | 500 /= per kg | 10,000 /= |
| Green pepper | 2 kg | 1,000 /= per kg | 2,000 /= |
| Carrots | 7 kg | 2,500 /= per kg | 17,500 /= |
| Cooking oil | 5 litres | 3,000 /= per litre | 15,000 /= |
| Charcoal | ½ sack | 20,000 /= per sack | 10,000 /= |
| Spices | 10 bags | 500 /= per bag | 5,000 /= |

1. What is the shopping list for?

__________________________________________________________________

1. How many items are shown?

__________________________________________________________________

1. What quantity of cooking oil will be bought? __________________________________________________________________
2. What is the price per unit of one kg of Irish potatoes?

__________________________________________________________________

1. What is the total amount of money that will be spent on tomatoes? __________________________________________________________________
2. What is the most expensive item on the list in terms of price per unit? __________________________________________________________________
3. What quantity of spices will be bought? __________________________________________________________________

**READ THE STORY AND ANSWER THE QUESTIONS BELOW IN FULL SENTENCES.**

Juma is 12 years old. He has four brothers and three sisters. Everyone in Juma’s family helps around the house, except for his youngest sister, Mary, who is only two years old and is too young to work. Juma’s favorite thing to do is work in the garden because he likes to see things grow. He is growing tomatoes, cassava and beans. He gets up very early in the morning to go to the garden. Juma also likes to go fishing with his brother. They sell what they can catch at the market.

1. How old is Juma?

…………………………………………………………………………………………

**35.** Who is Mary?

…………………………………………………………………………………………

**36.** What is Juma’s favorite thing to do?

…………………………………………………………………………………………

**37.** What does Juma like to do with his brother?

…..….………………………………………………………………………………….

**Innovations for Poverty Action: Project 79**

**ID**

**B**

**FOLLOW UP EXAM Code**

**TIME ALLOWED: 1 hr NAME: ___________________________**

**SCHOOL: _________________________**

**DATE: ___________________________**

***Section 1: Mathematics***

1. A factory produces 170 bars of soap per day and works 5 days a week. How many bars of soap will the factory produce by the end of one week?

________________________ bars of soap

1. 20 ÷ 4 = ____ ___ **3.** 7 - 10 = ____ __ _ **4.** 4 + -2 = __ __ ___

**5.** 14 **6.** 6369  **7.** 394 **8.** 512

x 4 - 3227 + 230 x 3

_____ _________ _______ ________

**9.**  ⅔ + ⅓ = __________ **10.** ⅝ - ⅜= _______ __

1. Moses has one 5,000 shilling note. He wants change for the same amount. Which of the following is the correct change? Circle the correct answer.
2. Two 1000 shilling notes, three 500 shilling coins, five 200 shilling coins
3. Three 1000 shilling notes, four 500 shilling coins
4. Four 1000 shilling notes, four 500 shilling coins
5. One 1000 shilling note, four 500 shilling coins, five 200 shilling coins
6. What is the time?


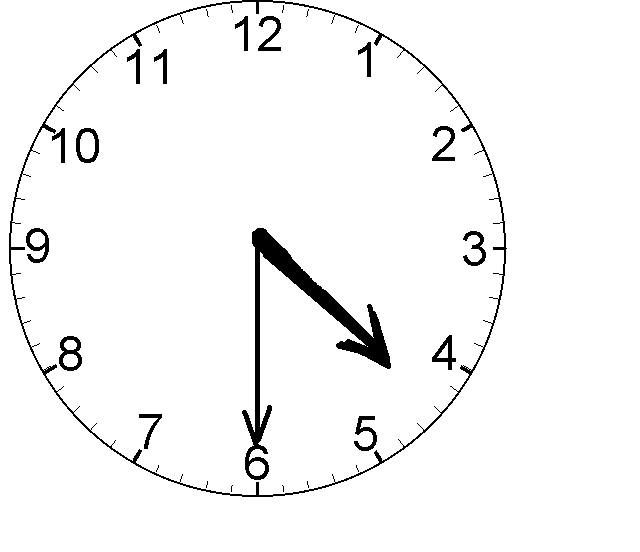
____________________

7 cm

1. Calculate the distance around the figure.

3 cm

Area: _____________

***Section 2: English***

**IN QUESTIONS 14-17, COMPLETE EACH SENTENCE WITH THE CORRECT FORM OF THE WORD IN BRACKETS.**

1. If you aren’t careful, the water _________________ on the ground. (spill)
2. Yesterday our teacher _____________ to the town council for a meeting. (go)
3. My older brother is much _____________ than I am. (tall)

**IN QUESTIONS 17-18, RE-WRITE EACH SENTENCE WITH THE OPPOSITE OF THE UNDERLINED WORD.**

1. Some plants will live if they are put in bright sunlight.

__________________________________________________________________

__________________________________________________________________

1. The store closes at 9 o’clock.

__________________________________________________________________

__________________________________________________________________

**IN QUESTIONS 19 AND 20, ARRANGE THE WORDS IN ALPHABETICAL ORDER.**

1. bucket, spent, nail, maize, house

__________________________________________________________________

1. return, rain, root, railway

__________________________________________________________________

**USE THE FOLLOWING WORDS TO MAKE A SENTENCE:**

1. we our for tomorrow study test will

_ ____________________________

1. very this raining morning started it early

_____________________________

***Section 3: English and Mathematics Combined***

**STUDY THE INFORMATION BELOW AND ANSWER THE QUESTIONS ABOUT IT IN FULL SENTENCES.**

**Shopping List for Auma’s Stall in the Market**

| **ITEM** | **QUANTITY AND UNIT** | **PRICE PER UNIT** | **TOTAL AMOUNT** |
| --- | --- | --- | --- |
| Tomatoes | 10 kg | 2,500 /= per kg | 25,000 /= |
| Onions | 10 kg | 1,000 /= per kg | 10,000 /= |
| Irish potatoes | 20 kg | 500 /= per kg | 10,000 /= |
| Green pepper | 2 kg | 1,000 /= per kg | 2,000 /= |
| Carrots | 7 kg | 2,500 /= per kg | 17,500 /= |
| Cooking oil | 5 litres | 3,000 /= per litre | 15,000 /= |
| Charcoal | ½ sack | 20,000 /= per sack | 10,000 /= |
| Spices | 10 bags | 500 /= per bag | 5,000 /= |

1. What is the shopping list for?

_________________________________________________________________

1. How many items are shown?

_________________________________________________________________

1. What quantity of cooking oil will be bought?

_________________________________________________________________

1. What is the price per kg of Irish potatoes?

_________________________________________________________________

1. What is the total amount of money that will be spent on tomatoes? _________________________________________________________________
2. What is the most expensive item on the list in terms of price per unit?

_________________________________________________________________

1. The total amount spent will be greatest for which item?

_________________________________________________________________

1. For how many items will 10 kg be bought?

_________________________________________________________________

**USE THE INFORMATION IN THE PASSAGE BELOW TO ANSWER THE QUESTIONS THAT FOLLOW.**

Godfrey’s mother is sick, so Godfrey is going to go to town to buy the things that she needs. First he will go to the market and then he will go to the pharmacy. Godfrey’s mother gave him a list of the things he needs to buy and how much they cost. He is going to buy things to make tea at the market and then to buy medicine for his mother at the pharmacy. He is also going to buy some oranges because they are very nutritious and good for people who are sick.

The cost of each of the items was:

1. milk: 700/= for one bag
2. sugar: 2,500 /= for one bag
3. tea: 2,000 /= for two bags
4. oranges: 4,000 for one kilogram
5. medicine tablets: 3,000 for one box

**ANSWER THE FOLLOWING QUESTIONS BASED ON THE PASSAGE ABOVE. CIRCLE THE LETTER THAT CORRESPONDS TO THE CORRECT RESPONSE.**

31. Where is Godfrey going to go?

a) Garden

b) Town

c) School

d) Medical clinic

32. Which place is Godfrey going to go first?

a) Pharmacy

b) Market

c) School

d) Medical clinic

33. How much will Godfrey spend on sugar and tea?

a) 4,500 /=

b) 3,000 /=

c) 700 /=

d) 2,700 /=

34. How much does a one bag of tea cost?

a) 1,000 /=

b) 500 /=

c) 700 /=

d) 2,000 /=

35. If Godfrey gave the pharmacist a 5,000 /= note to pay for the box of medicine, how much balance did the pharmacist give back to Godfrey?

a) 200 /=

b) 4,300 /=

c) 1,500 /=

d) 2,000 /=

36. What is Godfrey going to make with what he buys in the market?

a) A cake

b) Medicine

c) Coffee

d) Tea

37. Why is Godfrey going to buy oranges?

a) He likes how they taste

b) They are nutritious and good for people who are sick

c) They are his mother’s favorite fruit

d) They are in season

38. If Godfrey buys everything on his list, except for oranges, how much will he spend?

a) 2,000 /=

b) 7,500 /=

c) 8,000 /=

d) 8,200 /=
